# Transcriptomics in cancer revealed by Positron Emission Tomography radiomics

**DOI:** 10.1038/s41598-020-62414-z

**Published:** 2020-03-27

**Authors:** Florent Tixier, Catherine Cheze-le-Rest, Ulrike Schick, Brigitte Simon, Xavier Dufour, Stéphane Key, Olivier Pradier, Marc Aubry, Mathieu Hatt, Laurent Corcos, Dimitris Visvikis

**Affiliations:** 1 Department of Nuclear Medicine, Poitiers University Hospital, Poitiers, France; 2 LaTIM, INSERM, UMR 1101, Univ Brest, Brest, France; 3 Radiation Oncology Department, University Hospital, Brest, France; 4 INSERM, UMR 1078, Université de Brest, Génétique Génomique Fonctionnelle et Biotechnologies, Etablissement Français du Sang, Brest, France; 5 Head and Neck Department, Poitiers University Hospital, Poitiers, France; 6 CNRS, UMR 6290, IGDR, Université de Rennes 1, Rennes, France

**Keywords:** Cancer imaging, Tumour biomarkers, Tumour heterogeneity, Biomarkers

## Abstract

Metabolic images from Positron Emission Tomography (PET) are used routinely for diagnosis, follow-up or treatment planning purposes of cancer patients. In this study we aimed at determining if radiomic features extracted from ^18^F-Fluoro Deoxy Glucose (FDG) PET images could mirror tumor transcriptomics. In this study we analyzed 45 patients with locally advanced head and neck cancer (H&N) that underwent FDG-PET scans at the time of diagnosis and transcriptome analysis using RNAs from both cancer and healthy tissues on microarrays. Association between PET radiomics and transcriptomics was carried out with the Genomica software and a functional annotation was used to associate PET radiomics, gene expression and altered biological pathways. We identified relationships between PET radiomics and genes involved in cell-cycle, disease, DNA repair, extracellular matrix organization, immune system, metabolism or signal transduction pathways, according to the Reactome classification. Our results suggest that these FDG PET radiomic features could be used to infer tissue gene expression and cellular pathway activity in H&N cancers. These observations strengthen the value of radiomics as a promising approach to personalize treatments through targeting tumor-specific molecular processes.

## Introduction

^18^F-FDG Positron emission tomography (PET) imaging is largely used for diagnostic purposes in several cancer types, allowing accurate disease staging^1,2^, but it is also gaining ground for therapy applications, including monitoring treatment response and planning in the field of external beam radiotherapy^3^. Although visual interpretation may be sufficient for diagnosis, a (semi)quantitative analysis of PET data for image guided therapy applications is most frequently necessary. The simplest parameter usually extracted from PET images used in clinical practice is the maximum standardized uptake value (SUV_max_) corresponding to the highest single voxel intensity within a region of interest. On the one hand, SUV_max_ has often been reported as a biomarker with potential for improving overall patient management, including the prediction of response to therapy and survival in several cancers^4–6^. On the other hand, numerous studies have shown that the predictive/prognostic value of SUV_max_ can be limited, particularly when using images to characterize tumors on baseline PET images^7–9^. For this reason, there has been an increasing interest in extracting additional parameters from ^18^F-FDG PET images with the objective of more fully characterizing the entire tumor uptake. Most studies initially focused on the delineation of the metabolically active tumor volume (MATV) and associated SUV measurements (such as the mean value or total lesion glycolysis)^10,11^, while more recent studies have concentrated on parameters characterizing the shape or activity distribution within the tumor. These parameters, today known as radiomic features^14^, include 1^st^-order image features such as (cumulative) intensity histograms, geometrical tumor shape descriptors^12,13^, and higher order features aiming at quantifying intra tumor uptake heterogeneity (ITH)^8^.

The introduction of any new quantitative parameter derived from PET images in clinical practice raises several questions, including reproducibility, robustness, biological significance and potential impact on patient management. The reproducibility and robustness of radiomics with respect to overall image quality, reconstruction settings, partial volume effects and image segmentation have been evaluated in numerous studies, although almost exclusively for ^18^FDG^15–19^. Test-retest baseline studies have demonstrated an equivalent or better physiological reproducibility for a handful of radiomic features, compared to SUV_max_^19–21^. Finally, radiomics have shown potential for predicting the response to therapy or as prognostic factors of patient survival (including disease-free survival, overall survival or recurrence-free survival) in different cancer types^8,22,23^.

Despite these encouraging results, there are still numerous unanswered questions regarding the biological significance of PET radiomics, and their actual impact on overall patient management. It was initially hypothesized that intra-tumor ^18^FDG activity distribution heterogeneity may reflect tumor physiology, including tumor perfusion, glucose metabolism, hypoxia or angiogenesis^8^. In addition, anatomical radiomics (*i.e*., extracted from morphological computed tomography (CT) or magnetic resonance imaging (MRI) modalities) have been shown to correlate with other biological endpoints, including genomics, transcriptomics and metabolomics^24,25^.

A pioneering study of hepatocellular carcinomas showed that gene expression profiles could be associated to specific image traits from CT using a module network algorithm^26^. A recent study has shown the potential of CT radiomics to help recognizing the underlying gene expression patterns in lung and head-and-neck (H&N) cancer^27^. Importantly, it was shown that one oncogenic epidermal growth factor receptor (EGFR) mutation could be associated with ^18^FDG PET image features^28^. Within the same context, it would be informative to determine the relationships between transcriptomics and ^18^F-FDG PET radiomics, in order to obtain functional information relative to glucose metabolism, which most often represents a routine diagnosis and staging procedure in most cancers.

The main objective of this study was thus to investigate links between image radiomics and tumor-specific transcriptomics alterations. To this end, we conducted a pilot study to analyze data acquired prospectively from 45 H&N cancer patients. We characterized the transcriptome and extracted ^18^F-FDG PET radiomics from the same tumors. In order to identify thresholds of PET radiomic features that could be used to group the genes into co-regulated functional gene modules, both types of information were combined in a module network analysis. Finally, by identifying the pathways involved with those functional gene modules, we established a relationship between the values of PET radiomic features and altered cellular pathways.

## Patients and Methods

**Figure 1:**
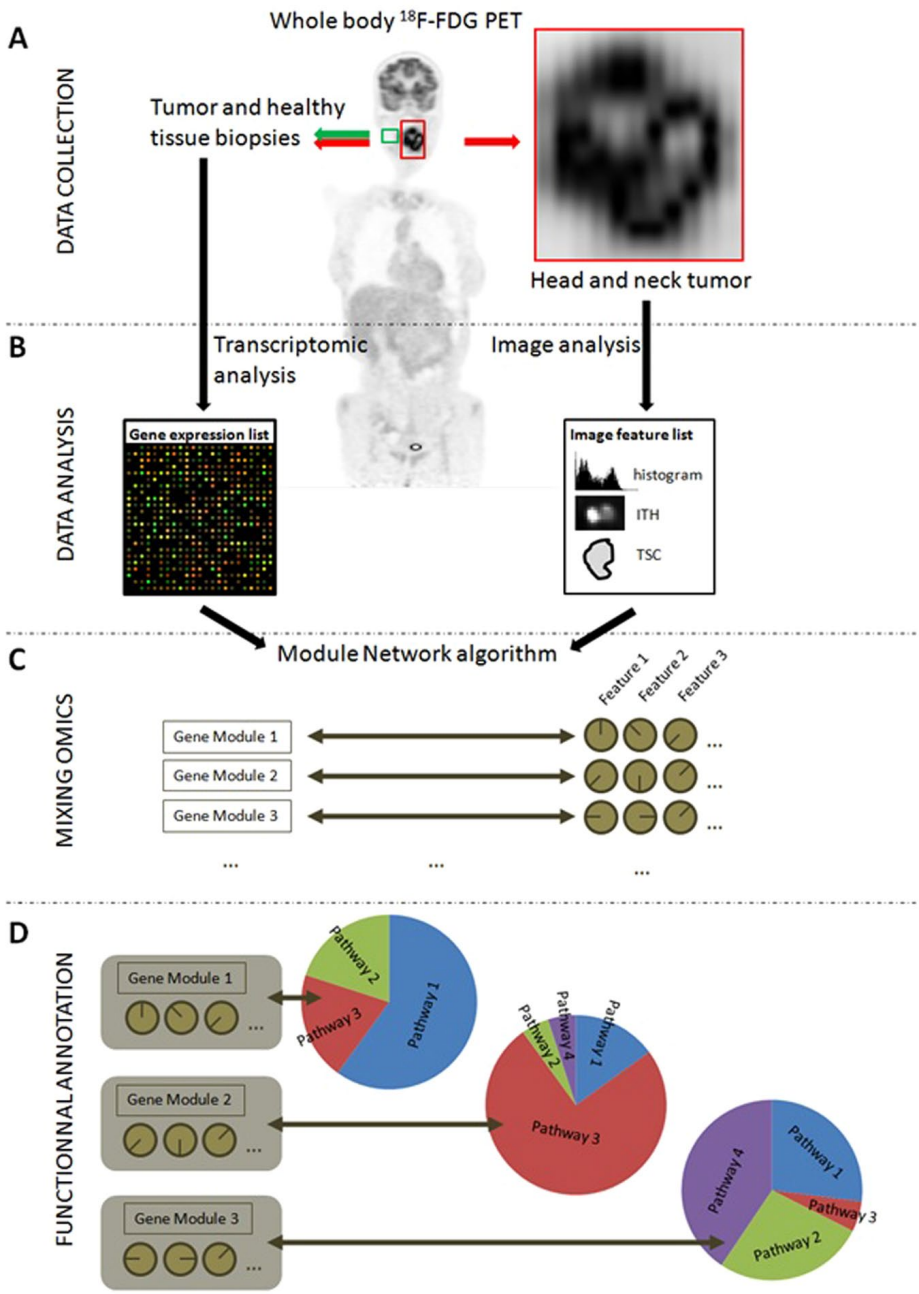
Global study workflow. (**A**) ^18^F-FDG PET acquisition and biopsies (both tumor and healthy tissues). (**B**) Data Analysis: RNAs were extracted from the surgical pieces and a transcriptome analysis was performed using Agilent 4 × 44 K microarrays. From PET images, tumors were automatically delineated and then PET radiomic features were calculated. (**C**) A module network algorithm was used to identify thresholds on PET features than could be used to split the gene list into modules of co-regulated genes. (**D**) The genes from each module were functionally annotated and placed into the main pathway to which they belonged. This last step allowed correlating altered pathways within gene modules and PET radiomic features.

The workflow of our analyses is presented in Fig. 1. It consists of four successive steps: data collection (A), transcriptomics and image analyses (B); mixing of transcriptomics and image data (C) and functional annotation (D).

### Patients

Forty-five patients with locally advanced H&N cancers were prospectively recruited since 2012 from two university hospitals  (Brest, France (n = 28); Poitiers, France (n = 17)). All patients were treated by chemoradiotherapy (platin based chemotherapy and 70 Gy with 2 Gy per fraction). Patient tumor fragments from both cancer and healthy tissues were biopsied for RNA preparation and transcriptome analysis with microarrays (Fig. 1A). All guidelines and regulations provided by the Brest University Hospital and the Poitiers University Hospital ethics review boards (ERBs) were followed during the experiments and all patients provided informed consent. The experimental protocol was also approved by these ERBs. Details of patients’ characteristics are given in Table 1.

**Table 1:** Patients’ characteristics.

**Gender**	
F	12
M	33
**TNM Staging**	
T2	4
T3	21
T4	20
N0	14
N1	8
N2	3
N3	19
M0	40
M1	5
**Stage**	
III	4
IV	41
**Histology**	
Squamous Cell Carcinoma	45
**Tumor Site**	
Hypopharynx	12
Larynx	3
Oropharynx	25
Oral Cavity	5

### FDG PET/CT image acquisition

All patients underwent an ^18^F-FDG PET/CT scan before initiating treatment as part of the routine staging procedure, within a maximum of 2 weeks from diagnosis. Patients fasted for at least 6 h and glucose levels were less than 10 mmol/L before injection of ^18^F-FDG (~5 MBq/kg), administered at 60 +/− 4 min before data acquisition. Data were acquired on a GEMINI PET/CT scanner (Philips, Cleveland, USA, n = 4) or a Biograph mCT40 (Siemens, Erlangen, Germany, n = 41). CT data were acquired first (120 kV and 100 mAs, no contrast enhancement). Three-dimensional PET images were reconstructed using CT-based attenuation correction and a 3-dimensional row-action maximum likelihood algorithm with a previously optimized protocol (2 iterations; relaxation parameter, 0.05; Gaussian post-filtering 5 mm; 4 × 4 × 4 mm^3^ voxels) for the GEMINI PET/CT scanner or an OSEM-TrueX-TOF (3 iterations; Gaussian post-filtering 5 mm; 4.07 × 4.07 × 2.03 mm^3^ voxels) for the Biograph mCT. SUVs were normalized using patient body weight.

### Radiomics analysis

Only the primary tumors were considered in this study and tumor delineation was performed using the Fuzzy Locally Adaptive Bayesian (FLAB) algorithm^10,15^. For comparison purposes, volumes obtained using a more widely available fixed threshold at 41% of the SUV_max_ were also generated. The MATV obtained through this method significantly underestimated the tumor uptakes in most of the images (mean volumes of 12 cm^3^ *vs*. 18 cm^3^ for fixed threshold at 41% of the SUV_max_ and FLAB respectively, p = 0.02), the radiogenomics analysis was only carried out by relying on the more accurate FLAB-derived tumor functional volumes (see Supplementary Fig. A and B for visual examples of the MATVs obtained using the two different segmentation approaches). The volume underestimation observed with the use of the 41% fixed threshold is in agreement with previous published studies^29–32^. This is most likely due to the highly irregular shapes in combination with the heterogeneous nature of uptake for the considered tumors.

A total of 28 image biomarker standardization initiative (IBSI)-compliant radiomic features were chosen^33^. This subset amongst existing radiomic features was selected in order to represent different categories of features (intensity, shape, textures of different scales), restricted to previously demonstrated reproducible ones^20,21^, as well as potentially predictive of outcome in H&N cancer^34,35^. The full list of extracted features and their formulas are listed in Supplementary Table 1.

For textural features from co-occurrence and intensity size-zone matrices, the fixed bin number (FBN) discretization was used with 64 bins. Texture matrices were constructed using relations between contiguous voxels along all 13 directions and all the intensity transitions were combined into one global matrix whatever the co-occurrence direction (merging strategy)^33^.

Shape descriptors included for example compactness, sphericity, surface to volume ratio and spherical disproportion^12,13^.

### Transcriptome analysis

A previously described method was used for the transcriptomic analysis^36^: H&N tissue samples were obtained from patients prior to any treatment. A surface fragment was collected from the cancer region, comprising on average 90% cancer cells, together with an adjacent fragment with no signs of dysplasia. The tissue fragments were then stored in RNAlater (Ambion, France). DNA and total RNA were extracted with the AllPrep DNA/RNA Mini kit (Qiagen, Courtabœuf, France) from homogenized tissue samples, according to the manufacturer’s instructions. RNA purity and integrity were determined by measuring the optical density ratio (A260/A280) and the RNA integrity number (RIN) was obtained using the RNA 6000 Nano LabChip (Agilent, Massy, France) and the 2100 Bioanalyzer (Agilent). Only RNA samples with a 28S/18S ratio > 1.0 and RIN ≥ 7.0 were used for microarray analyses.

An analysis of 45 RNA samples derived from H&N cancer tissues, matched with 45 samples from the same individuals recovered from non-tumor regions, was performed on 44 K Whole Human Genome microarrays (Agilent) that contain 41093 probes, providing full coverage of human transcripts. Double-stranded cDNA was synthesized from 500 ng of total RNA using the Quick Amp Labeling kit, One-color, as instructed by the manufacturer (Agilent). Labeling with cyanine3-CTP, fragmentation of cRNA, hybridization, and washes were performed according to the manufacturer’s instructions (Agilent). The microarrays were scanned and the data were extracted with the Agilent Feature Extraction Software.

The limma R package^37^ was used to preprocess and normalize the data: a background correction for each spot using the normexp method was first performed (adjusting the foreground adaptively for the background intensity of each spot), using a quantile between-array normalization. Values for replicate probes were then replaced with their average using the avereps function. The linear modeling function of limma was finally used to compute statistics for each probe and identify genes differentially expressed between conditions. Control of the false discovery rate (1%) was performed using the Benjamini-Hochberg procedure.

### Combination of transcriptomics data and PET features

The combination of transcriptomics and PET radiomics data was performed with the module network algorithm of Genomica software (https://genie.weizmann.ac.il/). This algorithm is based on probabilistic graphical models and was originally proposed to identify modules (*i.e*., groups of genes) of co-regulated genes, their regulators (such as transcription factors) and the conditions under which the regulation occurred^38^. It is an expectation-maximization (EM) algorithm^39^ that is used with a Bayesian scoring^40^ to identify modules of genes with a similar variation of the genes expression among the entire cohort of patients and combinations of PET radiomics that could explain these variations (Fig. 1B,C). Genomica was used with a maximum number of iterations of 30 and a maximum tree depth of 2, in order to avoid generating too small subgroups of patients. A module can be associated to up to 3 PET radiomic features, thus allowing for a maximum of 4 subgroups (see Supplementary Fig. C) to be constructed by the algorithm. Adaptation of this algorithm allowing the use of image characteristics as regulators was proposed before^26^ Radiomic features were normalized between 0 and 1 before being entered as input to Genomica. Consequently, thresholds obtained by Genomica were expressed as a percentage. For example, a threshold <5% indicates a value below 5% of the range between the minimum and maximum value of this feature. Because features are usually not normally distributed, this does not infer that a cut-off at 5% will necessarily provide a group containing only 5% of the patients.

As a final step, the identified gene modules were functionally annotated and positioned within the main biological pathways using the Reactome pathway database^41^.

## Results

**Figure 2:**
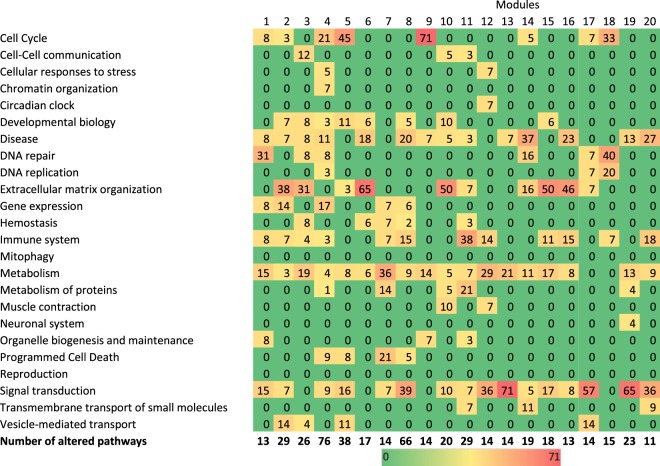
Percentage of significantly altered pathways that were involved in the main pathways of the Reactome Pathway Database. Only modules with more than 10 altered pathways are mentioned.

Upon comparing H&N samples to normal ones (12 women, 33 men; mean age 60.6, range [40; 76], Table 1, Fig. 1A), a total of 3315 probes were identified from the transcriptomic analysis (Fig. 1B) with a p_BH_ < 0.01 and a fold-change > 2 between the tumor biopsy/area and the adjacent macroscopically normal biopsy/area (with no signs of dysplasia, further verified by the pathologist). These probes corresponded to 1411 genes known in the Reactome database (42.6%). The use of these 3315 probes and the 28 PET radiomic features (Supplemental Table 1) in the module network algorithm allowed identifying thresholds enabling PET parameters to separate genes into distinct modules: 42 distinct modules could be associated with up to 3 PET radiomic features (Fig. 2). Among these 42 modules, 2 describe intra-correlations between the PET radiomic features themselves and not with the genes’ expression.

The 40 other modules, which contained 22–259 (mean 82 ± 57) probes, were constructed based on constraints using ^18^F-FDG PET features that are summarized in Table 2. The use of the probes from these modules in the Reactome Pathway Database allowed identifying altered pathways (Fig. 1D). The fraction of probes corresponding to known genes in the Reactome Pathway database varied from 16.7% to 70.8% (mean 40.6 ± 10.7%) as a function of the module (Supplementary Fig. D).

**Table 2:** Association between genes modules and 18F-FDG PET features.

Module #	Regulator 1		Regulator 2		Regulator 3		Groups size
1	Irregularity^‡^	29.5%	Angular second moment^†^	6.0%	Inverse difference moment^†^	5.9%	[14|10][05|16]
2	Irregularity^‡^	27.8%	Angular second moment^†^	5.8%	High-intensity large area emphasis^†^	22.8%	[11|11][07|16]
3	Irregularity^‡^	27.0%	Maximum distance to background^‡^	22.5%	Maximum distance to background^‡^	5.7%	[15|16][09|14]
4	Irregularity^‡^	29.5%	Angular second moment^†^	6.5%	MATV	4.9%	[14|11][05|15]
5	Intensity variability^†^	25.9%	Large area emphasis^†^	15.4%	—	—	[16|23][06]
6	SUV_COV_	79.2%	Maximum distance to background^‡^	5.7%	—	—	[15|24][06]
7	Angular second moment^†^	18.3%	Compactness v2^‡^	25.2%	High-intensity large area emphasis^†^	26.5%	[10|24][07|04]
8	Zone percentage^†^	77.3%	Inertia^†^	14.0%	—	—	[08|31][29]
9	ratio 3ds vol^‡^	25.5%	Maximum distance to background^‡^	22.5%	Large area emphasis^†^	15,4%	[11|07][09|18]
10	Size-zone variability^†^	6.6%	Angular second moment^†^	19.7%	High-intensity emphasis^†^	25.7%	[07|04][06|28]
11	Irregularity^‡^	27.8%	Large area emphasis^†^	26.9%	Dissimilarity^†^	71.8%	[12|10][17|06]
12	Irregularity^‡^	29.5%	Angular second moment^†^	5.9%	Inverse difference moment^†^	5.9%	[11|14][05|15]
13	Intensity variability^†^	25.9%	Maximum distance to background^‡^	5.7%	—	—	[15|24][06]
14	Compactness v2^‡^	86.5%	Large area emphasis^†^	21.7%	—	—	[20|19}[06]
15	Homogeneity^†^	18.6%	SUVmax	5.5%	Inverse difference moment^†^	20.0%	[08|08][05|24]
16	High-intensity large area emphasis^†^	19.3%	Large area emphasis^†^	20.3%	Intensity variability^†^	6.1%	[07|05][13|20]
17	Irregularity^‡^	27.8%	Large area emphasis^†^	28.0%	Maximum distance to background^‡^	18.1%	[12|10][11|12]
18	Intensity variability^†^	25.9%	Large area emphasis^†^	15.4%	—	—	[16|23][06]
19	Inertia^†^	20.6%	—	—	Large area emphasis^†^	27.7%	[16] [25|04]
20	Inertia^†^	20.6%	SUV_COV_	34.4%	Zone percentage^†^	73.1%	[07|09][16|13]

The cut-off from regulator 1 splits patients into two groups, that are then split using the regulator 2 and 3. Regulator 2 and 3 are applied to the groups of patients with a value < and > = the threshold on the regulator 1, respectively. The values are expressed as percentage based on the distribution within the cohort. ^†^Heterogeneity feature, ^‡^shape feature.

Values for each regulator correspond to the cut-off between the two groups.

The number of altered pathways in cancer samples ranged between 0 and 76 (mean 14.5 ± 15.8), depending on the considered module. There was no statistically significant correlation between the number of genes within a given module and the number of altered pathways.

Genes from 20 modules were involved in more than 10 significantly altered pathways. When these pathways were grouped into the 24 main pathways in the Reactome hierarchy, modules produced with Genomica were composed of genes that were already described in the cell-cycle, disease, DNA repair, extracellular matrix organization, immune system, metabolism or signal transduction pathways (Fig. 2).

**Figure 3:**
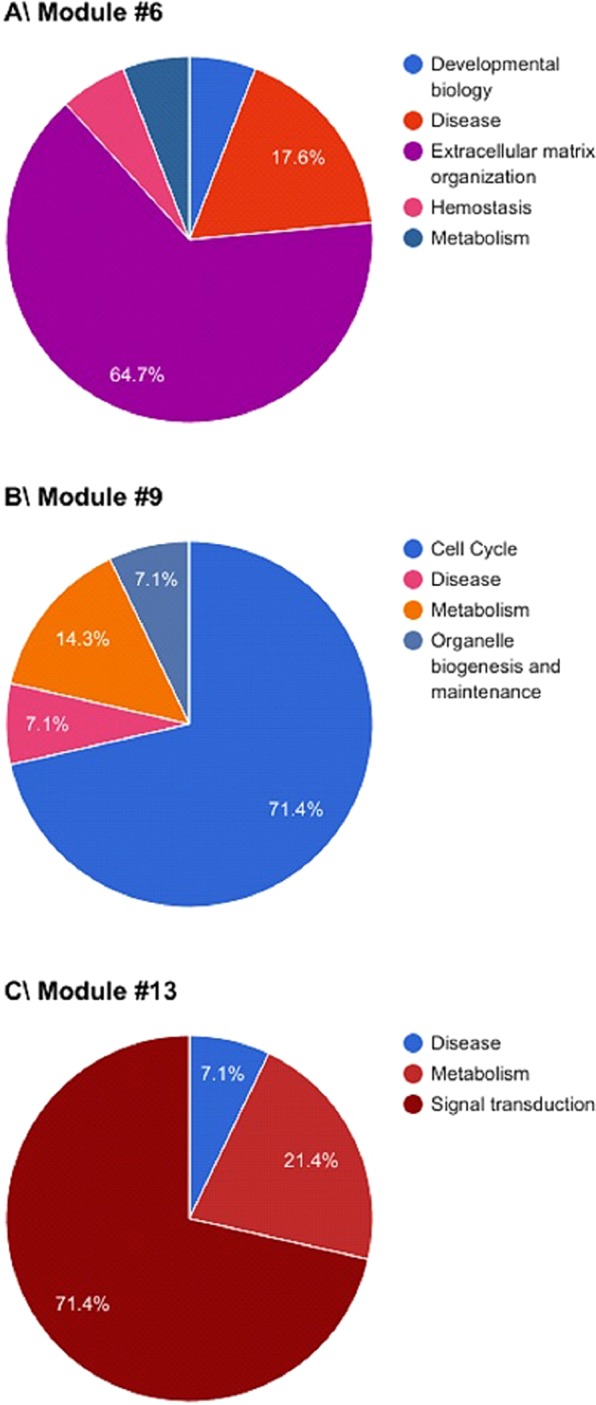
Gene modules identified by Genomica that were involved in more than 50% in (**A**) extracellular matrix organization, (**B**) cell cycle and (**C**) Signal transduction.

**Figure 4:**
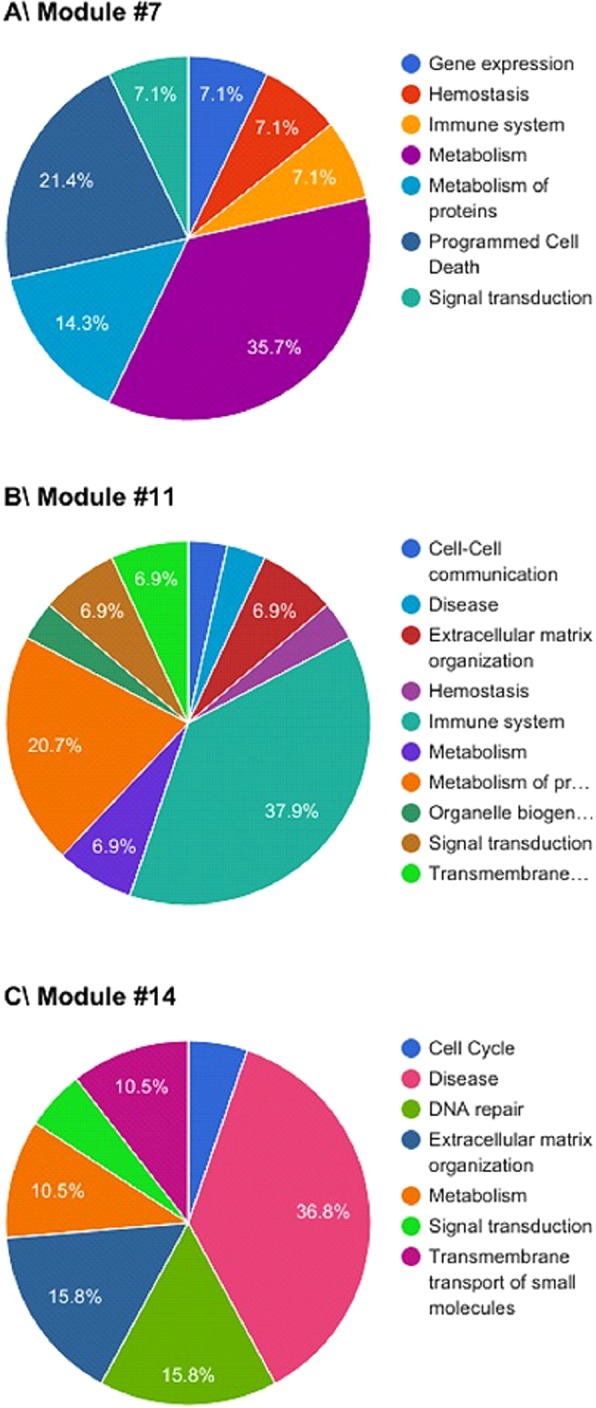
Gene modules identified by Genomica that were involved in more than 30% in (**A**) metabolism, (**B**) immune system and (**C**) disease.

Seven modules had more than 50% of their significantly altered pathways involved in the same main Reactome pathway as pictured in the first case of Fig. 1D. These modules were mostly associated with extracellular matrix organization (modules #6, Fig. 3A (65%); #10 (50%) and #15 (50%)), cell cycle (module #9, Fig. 3B (71%)) or signal transduction (modules #13, Fig. 3C (71%); #17 (57%) and #19 (65%)). Modules not presented in Fig. 2 can be found in the Supplemental Material. Twelve other modules had at least 30% of their significantly altered pathways involved in the same main Reactome pathway, including metabolism (module #7, Fig. 4A (36%)), the immune system (module #11, Fig. 4B (38%)), disease (module #14, Fig. 4C (37%)), cell cycle (module #18 (33%)), DNA repair (modules #1 (31%) and 18 (40%)), extracellular matrix organization (modules #2 (38%); #3 (31%) and #16 (46%)) or signal transduction (modules #8 (39%); #12 (36%) and #20 (36%)). Modules not presented in Fig. 4 can be found in the Supplemental Material. One module (#4, Fig. 2) was not found to be associated with any particular Reactome pathway. However, significant associations were found between genes from this module and 10 main Reactome pathways (Supplementary Fig. D).

These associations between PET radiomic features and altered biological pathways pointed to a direct link between the two. For instance, the alteration of extracellular matrix organization, which could be identified by modules #2, #3, #6, #10, #15 and #16 (Table 2), was associated with a threshold of 28% on the irregularity, followed by a threshold of 6% on the angular second moment and a threshold of 23% on the high-intensity large area emphasis for module #2; with a threshold of 27% on the irregularity followed by a threshold of 6 and 23% on maximum distance to background for module #3; with a threshold of 79% on SUV_cov_ followed by a threshold of 6% on maximum distance to background > 6% for module #6; with a threshold of 7% on size-zone variability followed by a threshold of 20% on angular second moment and a threshold of 26% on high intensity emphasis for the module #10; with a threshold of 19% for the homogeneity followed by a threshold of 5% on SUV_max_ > 5% and a threshold of 20% on inverse difference moment for the module #15; and with a threshold of 19% on high-intensity large area emphasis followed by a threshold of 20% on large area emphasis and a threshold of 6% on intensity variability for the module #16 (Table 2).

Some ^18^F-FDG PET radiomic features, such as angular second moment and inverse difference moment, two local heterogeneity parameters, or large area emphasis and intensity variability, two regional heterogeneity, or irregularity and maximal distance to background, two shape features, were most frequently selected by the Genomica module network algorithm.

## Discussion

The central assumption of our approach was that the alteration of gene expression involved in specific biological pathways was the driver of altered PET radiomics, but not the opposite. Our aim was thus to determine, using this combined “radiomics – transcriptomics” process and starting from the sole analysis of PET images, if it would be possible to approach, in a realistic way, the biological tumor characteristics without conducting a comprehensive molecular analysis of each tumor. This would help selecting the best curative approach more rapidly, while minimizing costs and time to prescription.

In 2010, radiomics was defined as the extraction of quantitative features from radiographic images^42^ and then extented in 2012 to the high-throughput extraction of large amounts of image features from medical images^24^. This was linked with the concept of radio-genomics whereby radiomics could be used to understand the relationship between image features and biological characteristics^24,43^. Several studies have investigated optimization of radiomic features extraction^12,43–45^. Other studies have focused on building radiomic models predictive of a clinical endpoint, such as survival or response to therapy^46,47^. To date, only a few studies have investigated the functional links between radiomics and other omics data^26,27,48^. In 2007, Segal *et al*. have suggested that gene expression profiles could be reconstructed using “image traits” derived from CT scans in hepatocarcinoma^26^. In these and other studies, radiomic features derived from CT images could be associated with genes expression/annotation^26,27^. In addition, these radiomics were demonstrated to be cancer-specific^49^, providing strong evidence that anatomical image-derived features could highlight the underlying biology.

Our study aimed at exploring if and how ^18^F-FDG PET image derived radiomics could be used to identify differences in gene expression, for a given patient, between cancer and healthy tissues, and to see if we could also recognize distinct profiles between patients that had the same disease but potentially different prognoses. Recent studies have shown the potential of radiomics to provide signature to predict results on molecular biomarkers. For instance, it was showed that some specific mutations, such as EGFR or KRAS mutations, could be identified on baseline ^18^F-FDG PET scans in non-small cell lung cancer^28^. In H&N cancer, the change of the tumor-to-background ratio at baseline and after two weeks of chemoradiotherapy in ^18^F-MISO PET images can be correlated with hypoxia biomarkers (HIF1α and CAIX)^50^. In colorectal cancer, radiomics features were correlated with the expression changes of *ABBC2*, *CD166*, *CDKNV1*, and *INHBB* genes^51^. A radiomic signature of the tumor infiltration (CD8) was identified and validated on patients (multi-cancer site) treated with anti-PD-1 or anti-PD-L1^52^. Only one recent study has used data from the Cancer Genome Atlas (TCGA) to correlate radiomic signatures with molecular functions using the whole transcriptomic expression, in bladder urothelial carcinoma^53^. To our knowledge, our study represents the first attempt to investigate the associations between ^18^F-FDG PET radiomics and the whole transcriptome expression in a set of prospectively recruited H&N cancer patients. Our results showed that expression of co-regulated genes from distinct biological pathways was correlated with selected FDG PET radiomic features. They also suggest that while some standard 1^st^ order intensity measurements such as SUV_cov_ can help identifying subsets of patients with alterations in the extracellular matrix organization, the addition of shape and higher order radiomic features can allow the discovery of many more associations between FDG PET images and biological pathways’ alterations. This could likely reflect tumor heterogeneity, which is quite clear at the molecular level in H&N^54^. Such an ability to differentiate the patients according to radiomics, together with inferring on the activity of molecular pathways, could have a strong impact on the quality of the prediction of the response to treatment and the recurrence-free survival^54,55^. Using this heterogeneity as a basis for a more personalized treatment is a very appealing perspective that holds a high potential benefit for patients^54^. In H&N squamous cell carcinoma, four subtypes of cancers were identified using expression profiling techniques^56^. Our study suggests that these subtypes, and potentially others, could be directly identified using baseline ^18^F-FDG PET images currently acquired as part of the diagnostic management of HNC patients, as it has been shown similarly from CT scans^55^. Indeed, according to the values of the PET radiomic features, it would be possible to position a patient on a specific configuration in the identified modules, and from that, infer potentially altered biological pathways. In contrast to previously published studies, we did not aim at linking radiomic features to genetic biomarkers that had previously been identified. Our findings suggest that radiomic signatures usually identified with the goal of outcome prediction, could also be used to identify transcriptomic profiles. Revealing transcriptomics through radiomics may find multiple applications, including (i) identify patients that could benefit from personalized therapy, (ii) discover potential targets for the design of new treatments, (iii) provide a biological meaning for the radiomic signatures as well as the possibility to test a signature through biological biomarkers. This last point may largely help in moving an additional step towards a potential clinical usage of radiomics.

The novelty of the approach described in this proof-of-concept study is not compromised by certain limitations. Firstly, we designed this prospective study to investigate the ability of radiomic features to reflect biological processes as an exploratory study and exploited the entire prospective cohort of 45 patients for that purpose. We have therefore not further evaluated our findings in a validation cohort. However, the patients included in this study were recruited prospectively. The collection of such a validation cohort will clearly require more time and have financial implications given the whole transcriptome analysis required per patient. Our aim will be to start in the future the acquisition of such an internal validation cohort. External validation using similar datasets collected by other teams will also be considered. Second, we decided to limit the number of radiomic features included in the analysis, to make the radiomic part of the study as simple but also as robust as possible: we have (1) used only one discretization approach with one parameter (FBN, 64 bins), (2) kept the original image definition (no interpolation), (3) considered one robust segmentation method (FLAB^10^) and no other alternative solution^29^, and (4) investigated only some of the existing features, chosen to be representative of the different categories of features (intensity, texture and shape) and selected based on their previously demonstrated level of test-retest reproducibility^19,20^ and potential value in predicting outcome in H&N cancer^34,35^. Combinations of different preprocessing options and a more advanced features selection, such as LASSO^57^, could potentially improve our results.

Two different scanner models and reconstruction algorithms were used in the acquisition of the PET images. However, only 4 patients were acquired on the first scanner model, whereas the majority (n = 41) were acquired on the other, thus reducing the risk of introducing a bias in that respect. Additionally, the radiomic features (chosen to be robust and reproducible) distributions of these 4 patients did not deviate significantly from those of the 41 other patients.

Future work will include analysis of the low-dose CT images from the PET/CT acquisitions and prediction of outcome in the patients using combined radiogenomics. Further prospective studies should help determining if straight radiomics, such as those using ^18^F-FDG PET, could be used to select the most appropriate and personalized treatment by targeting tumor-specific molecular processes, either alone or in combination with some other disease marker, easily captured by other non-invasive methods like liquid biopsies.

## Supplementary information

Supplementary information.
