# Supplementary material for: Transcriptomics in cancer revealed by Positron Emission Tomography radiomics
Source: Sci Rep. 2020 Mar 27;10:5660. doi: 10.1038/s41598-020-62414-z (PMC7101432; doi:10.1038/s41598-020-62414-z)
Supplement: Supplementary file 1 — Supplementary information. [file 41598_2020_62414_MOESM1_ESM.docx]

**Transcriptomics in cancer revealed by Positron Emission Tomography radiomics**

Supplementary Information

Florent Tixier, Catherine Cheze-le-Rest, Ulrike Schick, Brigitte Simon, Xavier Dufour, Stéphane Key, Olivier Pradier, Marc Aubry, Mathieu Hatt, Laurent Corcos, Dimitris Visvikis

**Radiomics features:**

B: binning value (64 in this paper)

N_v_: number of voxels in the tumor

N_z_: number of homogeneous areas in the tumor

Z_max_: the size of the largest homogenous areas in the tumor

C: co-occurrence matrix. The size of matrix C is BxB

GLSZM: Gray-level size-zone matrix. The size of GLSZM is BxZ_max_

C(i,j) or GLSZM(i,j) refers to the i^th^ line and j^th^ column of matrices C and GLSZM respectively

*Volume*

#1: MATV: Metabolic Active Tumor Volume

Defined using the fuzzy locally adaptive Bayesian (FLAB) algorithm ^1^

#2: TLG (Total Lesion Glycolysis)

Product between the MATV and the mean value of the Standardized Uptake Value in the MATV

$$TLG={MATV.SUV}_{mean}$$

*Histogram features*

#3 SUV_max_: Maximum value of the Standardized Uptake Value

#4 SUV_cov_: Covariance (mean divided by the standard deviation (SD)) of the Standardized Uptake Value in the MATV)

$${SUV}_{cov}=\frac{{SUV}_{mean}}{{SUV}_{SD}}$$

*Cumulative intensity histogram (CIH)*^2^

#5: CIH_AUC_: Area under the curve of the cumulative intensity histogram

*Co-occurrence features*^3^

#6: ASM: Angular Second Moment (e.g. Energy or Uniformity)

$$ASM=\sum_{i=1}^{B} \sum_{j=1}^{B} {C(i,j)}^{2}$$

#7: IDM: Inverse Different Moment

$$IDM=\sum_{i=1}^{B} \sum_{j=1}^{B} \frac{C(i,j)}{{1+(i-j)}^{2}}$$

#8: Entropy

$$Entropy=-\sum_{i=1}^{B} \sum_{j=1}^{B} C\left( i,j \right).ln\left( C\left( i,j \right) \right)$$

#9: Correlation

$$Correlation=\frac{\sum_{i=1}^{B} \sum_{j=1}^{B} \left( i-\mu_{x} \right)\left( j-\mu_{y} \right)C\left( i,j \right)}{\sigma_{x}\sigma_{y}}$$

With

$$\mu_{x}=\sum_{i=1}^{B} \left( i\sum_{j=1}^{B} C\left( i,j \right) \right);\mu_{y}=\sum_{j=1}^{B} \left( j\sum_{i=1}^{B} C\left( i,j \right) \right);$$

$$\sigma_{x}=\sqrt{\sum_{i=1}^{B} \left( \left( i-\mu_{x} \right)^{2}\sum_{j=1}^{B} C\left( i,j \right) \right)};\sigma_{y}=\sqrt{\sum_{j=1}^{B} \left( \left( j-\mu_{x} \right)^{2}\sum_{i=1}^{B} C\left( i,j \right) \right)}$$

#10: Homogeneity

$$IDM=\sum_{i=1}^{B} \sum_{j=1}^{B} \frac{C(i,j)}{1+\left| i-j \right|}$$

#11: Dissimilarity

$$Dissimilarity=\sum_{i=1}^{B} \sum_{j=1}^{B} \left| i-j \right|.C(i,j)$$

#12: Inertia (contrast)

$$Inertia=\sum_{i=1}^{B} \sum_{j=1}^{B} \left| i-j \right|^{2}.C(i,j)$$

*Gray-Level Size-Zone Matrix (GLSZM)-based features*^4^

#13: ZP: Zone percentage

$$ZP=\frac{N_{z}}{N_{v}}$$

#14: HIE (High Intensity Emphasis)

$$HIE=\frac{1}{N_{z}}\sum_{i=1}^{B} \sum_{j=1}^{Z_{max}} i^{2}.GLSZM(i,j)$$

#15: LAE (Large Area Emphasis)

$$LAE=\frac{1}{N_{z}}\sum_{i=1}^{B} \sum_{j=1}^{Z_{max}} j^{2}.GLSZM(i,j)$$

#16: HILAE (High-Intensity Large-Area Emphasis)

$$HIE=\frac{1}{N_{z}}\sum_{i=1}^{B} \sum_{j=1}^{Z_{max}} i^{2}.j^{2}.GLSZM(i,j)$$

#17: IV (Intensity Variability)

$$IV=\frac{1}{N_{z}}\sum_{i=1}^{B} \left( \sum_{j=1}^{Z_{max}} GLSZM(i,j) \right)^{2}$$

#18: SZV (Size-Zone Variability)

$$SZV=\frac{1}{N_{z}}\sum_{j=1}^{Z_{max}} \left( \sum_{i=1}^{B} GLSZM(i,j) \right)^{2}$$

*Shape features*

#19: MTS: Metabolic tumor surface. Calculated by summing the surface of the face of each voxel spatially connected with the background.

#20: Ratio_MTS_MATV (ratio between the 3D metabolic tumor surface and the metabolic tumor volume)

$$Ratio\_3ds\_vol=\frac{MTS}{MATV}{cm}^{-1}$$

#21: Ratio_3ds_vol_norm

$$Ratio\_3ds\_vol\_norm=\frac{MTS}{3.MATV}{\left( \frac{3.MATV}{4\pi} \right)^{\frac{1}{3}}cm}^{-1}$$

#22: Irregularity

$$\mathrm{Irregularuity}=\frac{\sum_{i=1}^{6} i.d(i)}{\sum_{i=1}^{6} d(i)}$$

With d(i) the number of voxels having I neighbor in the surrounding background

#23: d2b_max (maximal distance tumor of an internal tumor voxel to the background)

#24: tumor length: longest distance between two voxels within the tumor

#25: Compactness v1

$$Compactness v1=\frac{MATV}{{\sqrt{\pi}MTS}^{2/3}}$$

#26: Compactness v2

$$Compactness v1=36\pi\frac{{MATV}^{2}}{{MTS}^{3}}$$

#27: Sphericity

$$\mathrm{Sphericity}=\frac{\sqrt[3]{36\pi.{MATV}^{2}}}{3ds}$$

#28: Spherical disproportion

$$Sperical disproportion=\frac{MTS}{4\pi.\left( 3\frac{MTV}{4\pi} \right)^{1/3}}$$

References

1. Hatt, M., Rest, C. C. le, Turzo, A., Roux, C. & Visvikis, D. A Fuzzy Locally Adaptive Bayesian Segmentation Approach for Volume Determination in PET. *IEEE Trans. Med. Imaging* **28,** 881–893 (2009).

2. van Velden, F. H. P. *et al.* Evaluation of a cumulative SUV-volume histogram method for parameterizing heterogeneous intratumoural FDG uptake in non-small cell lung cancer PET studies. *Eur. J. Nucl. Med. Mol. Imaging* **38,** 1636–1647 (2011).

3. Haralick, R. M., Shanmugam, K. & Dinstein, I. Textural Features for Image Classification. *IEEE Trans. Syst. Man Cybern.* **SMC-3,** 610–621 (1973).

4. Thibault, G. *et al.* Texture indexes and gray level size zone matrix. Application to cell nuclei classification. in *10th International Conference on Pattern Recognition and Information Processing, PRIP 2009* 140–145 (2009).

**Genes Modules:**

Reactome pathways involved in each gene modules identified by Genomica. Modules #1-20 are the ones with more than 10 genes identified and Modules #21-40 are the ones with less than 10 genes. There is no graph for modules #31 and #35 because none of the genes for these modules were found involved in known pathways.


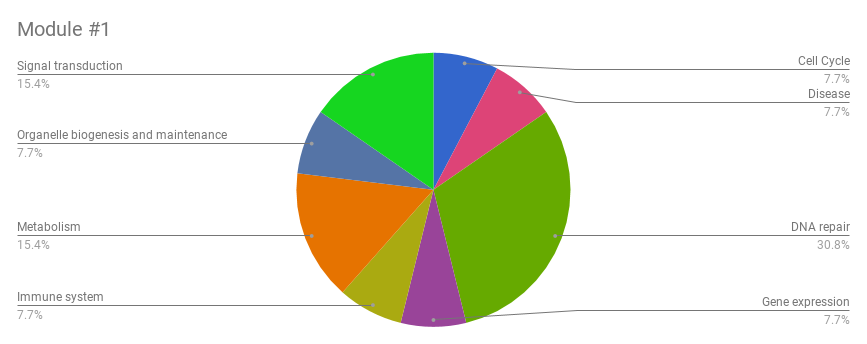

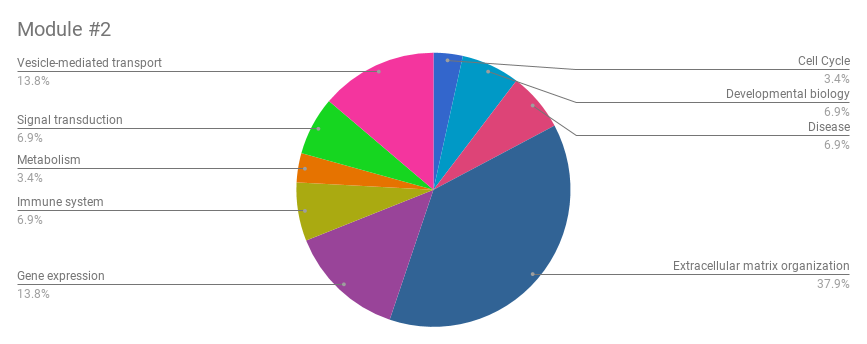

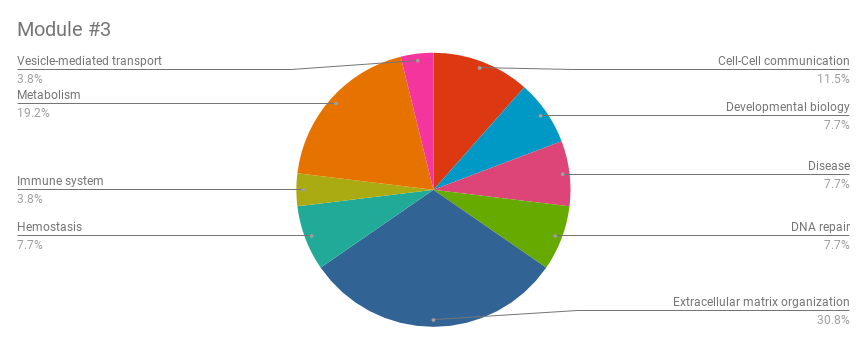

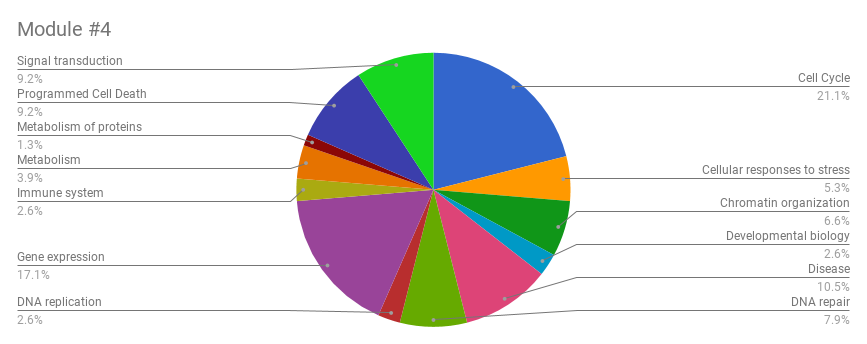


**
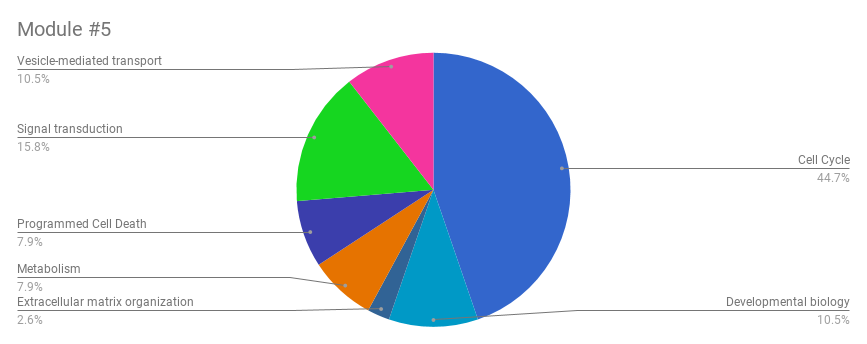

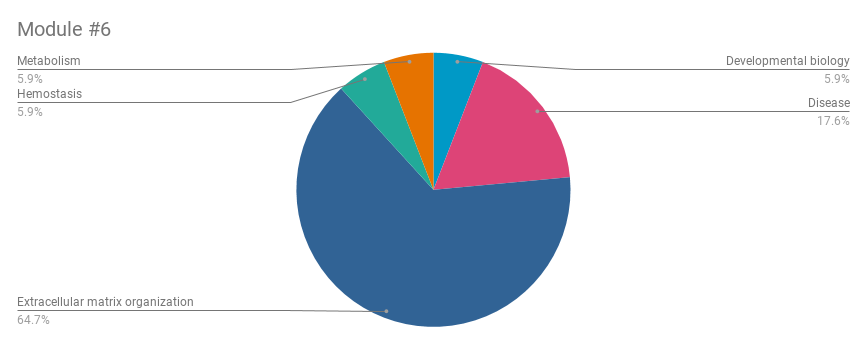
**

**
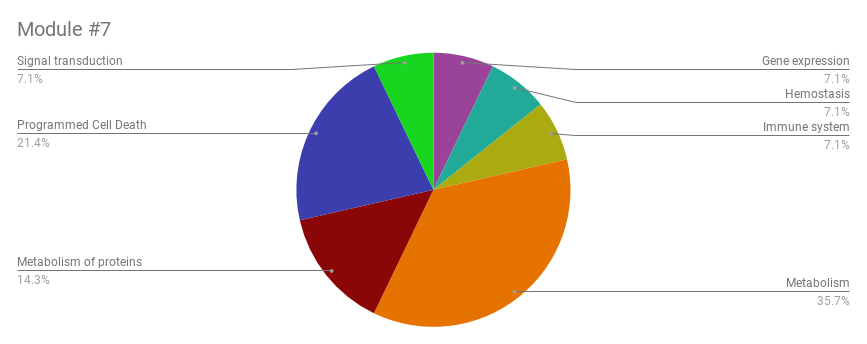
**

**
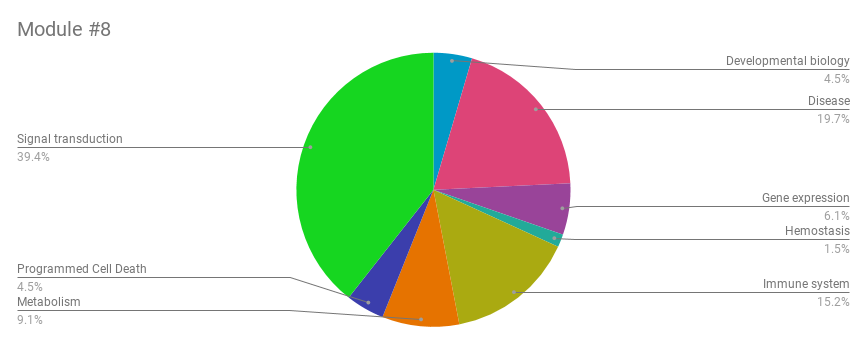
**

**
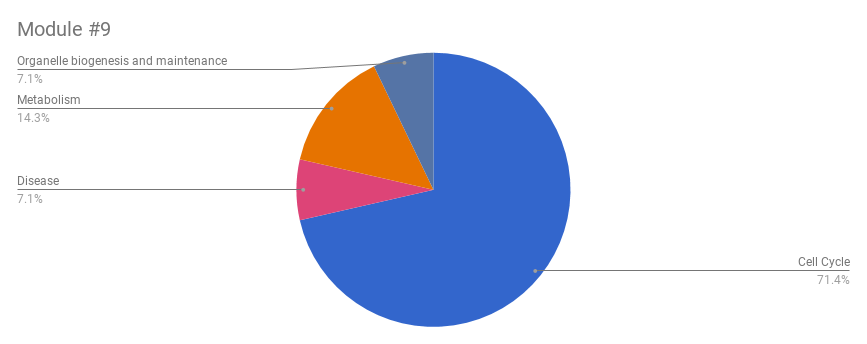
**

**
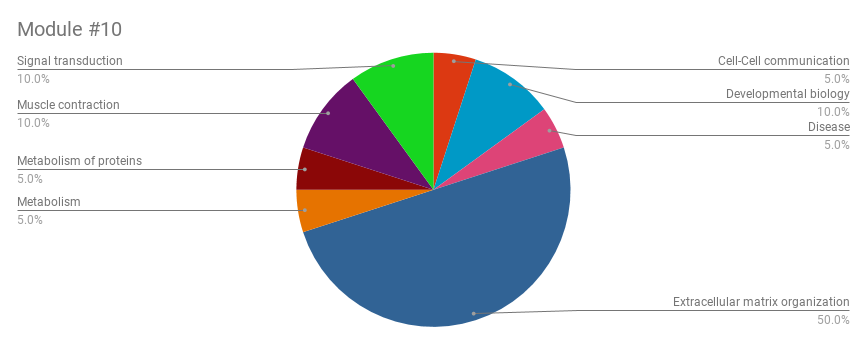
**

**
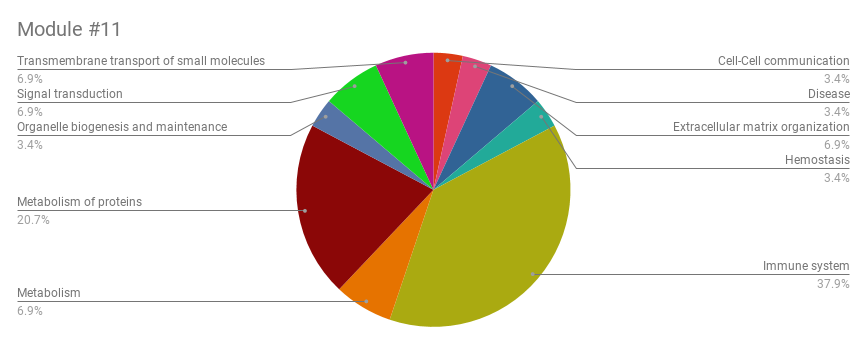
**

**
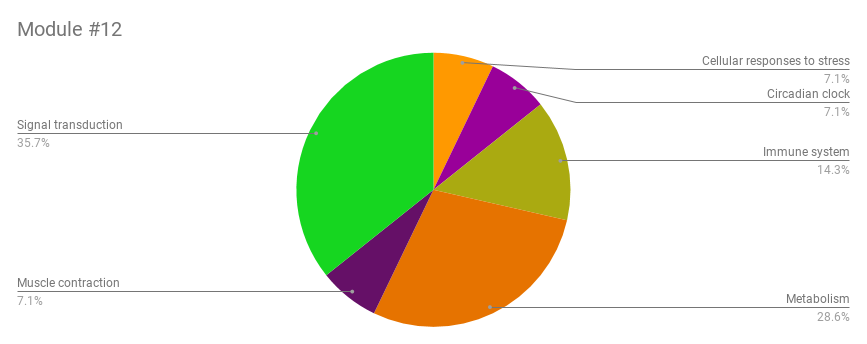
**

**
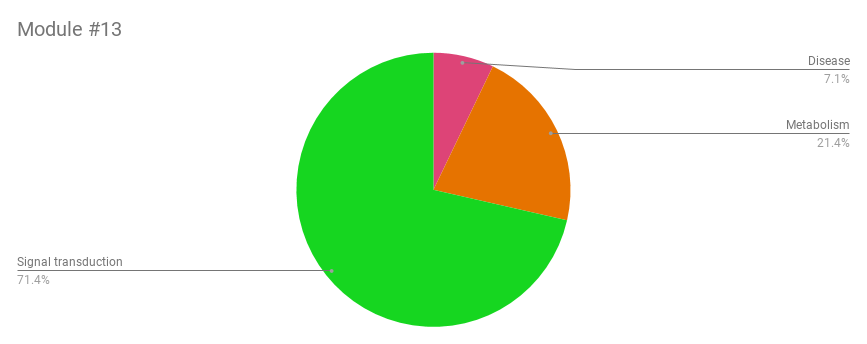
**

**
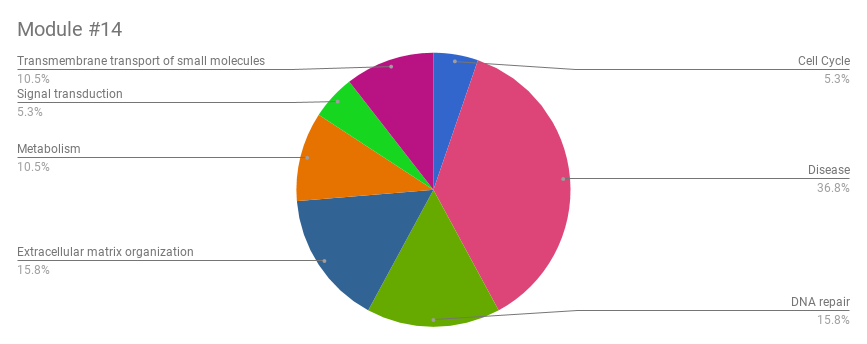
**

**
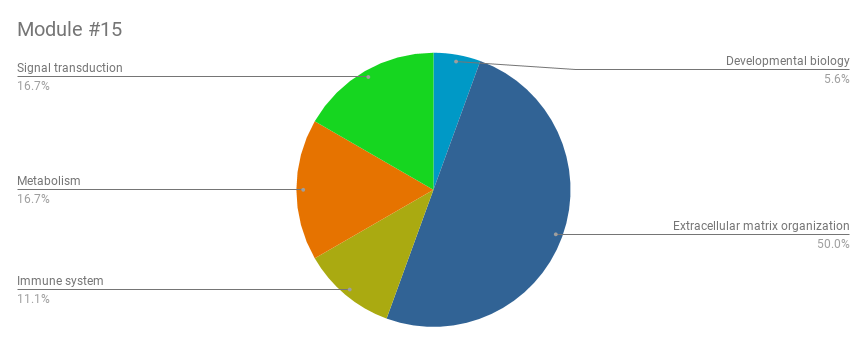
**

**
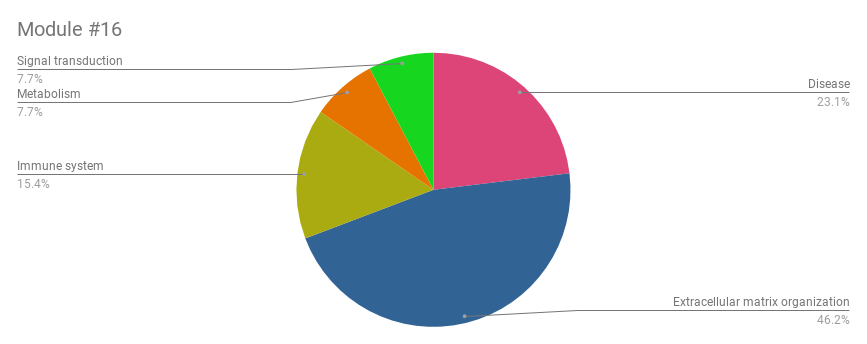
**

**
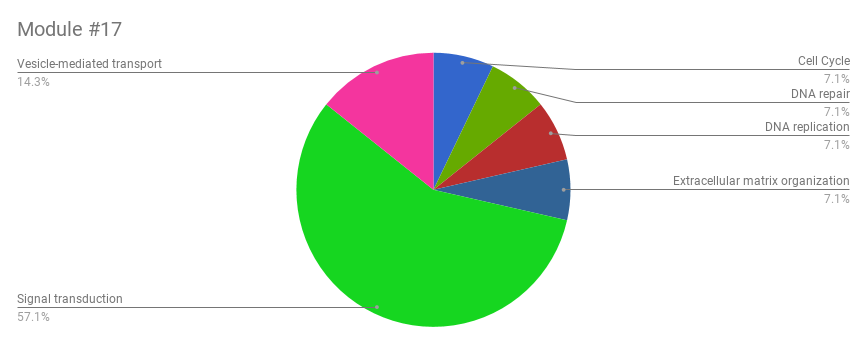
**

**
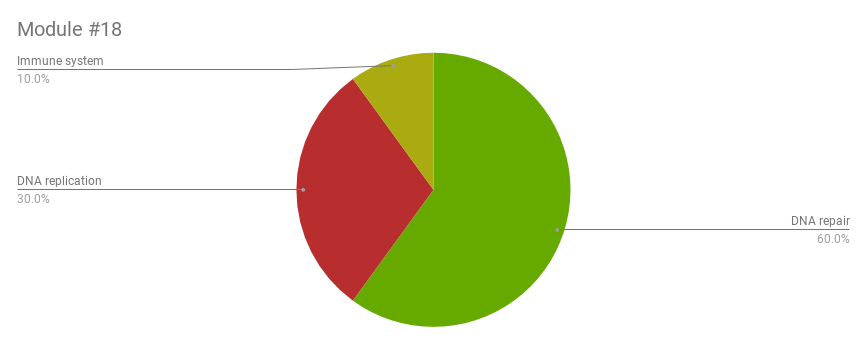
**

**
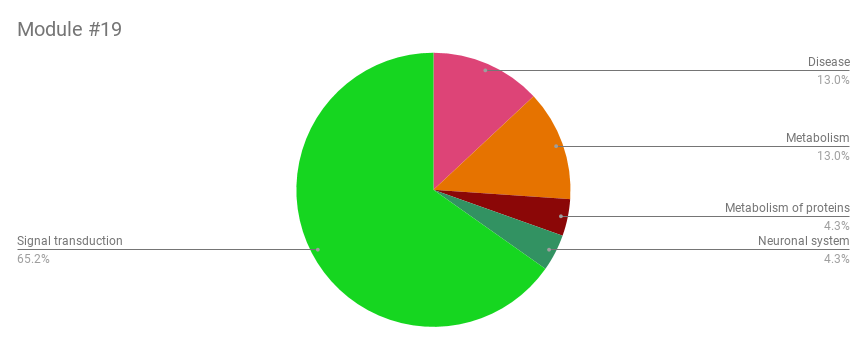
**

**
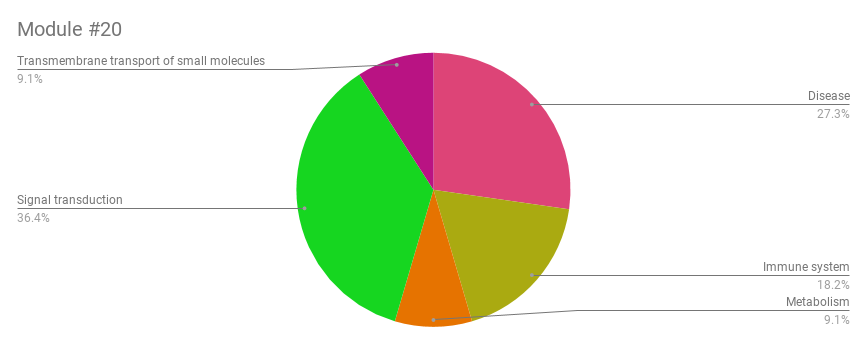
**

**
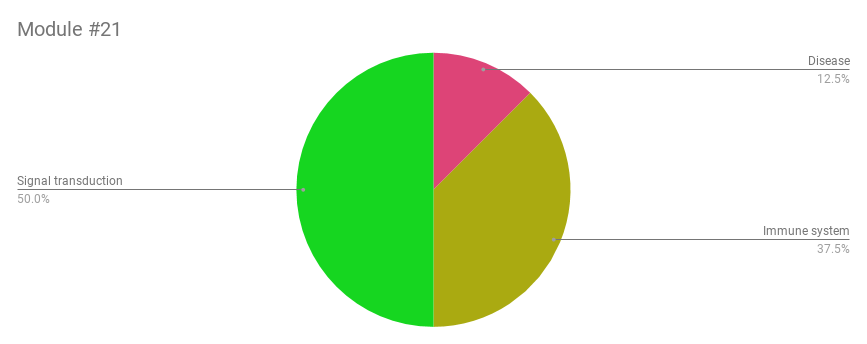
**

**
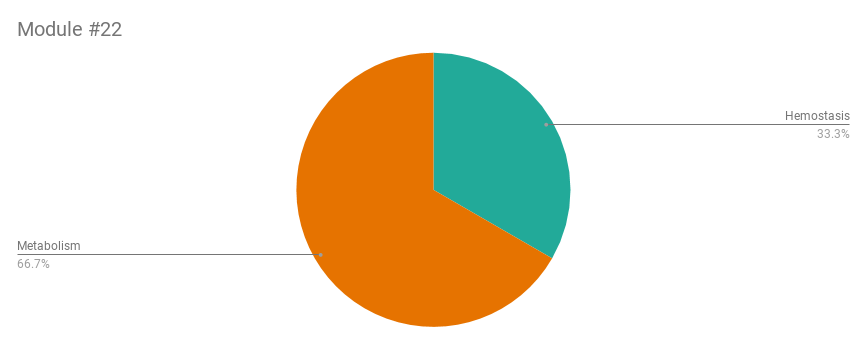
**

**
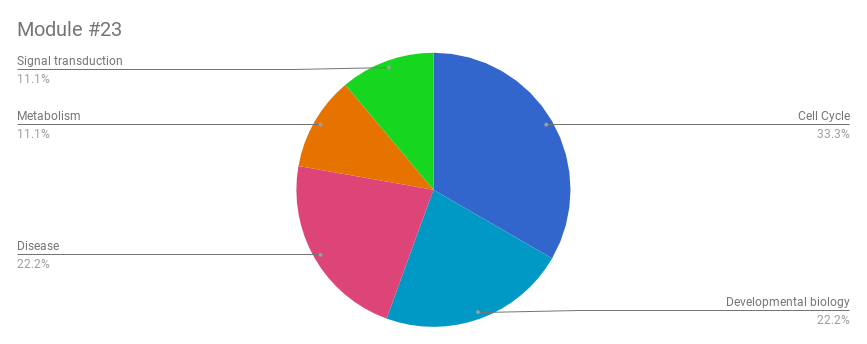
**

**
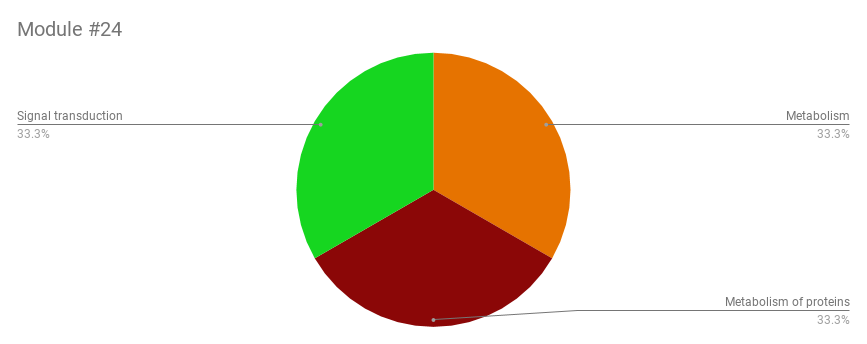
**

**
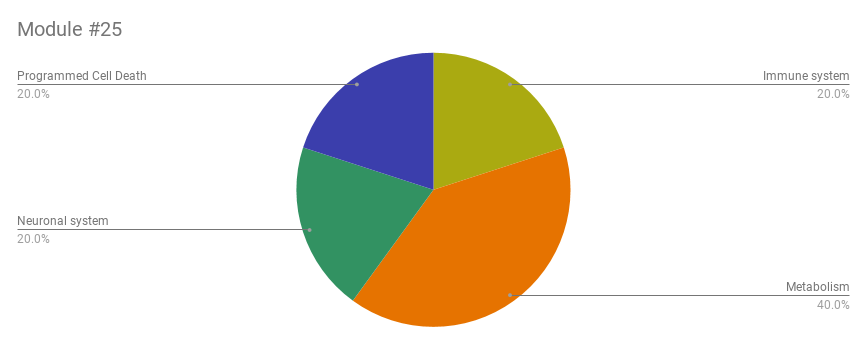
**

**
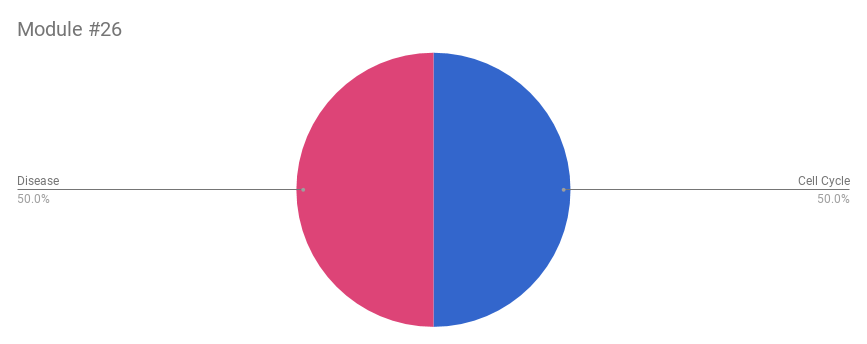
**

**
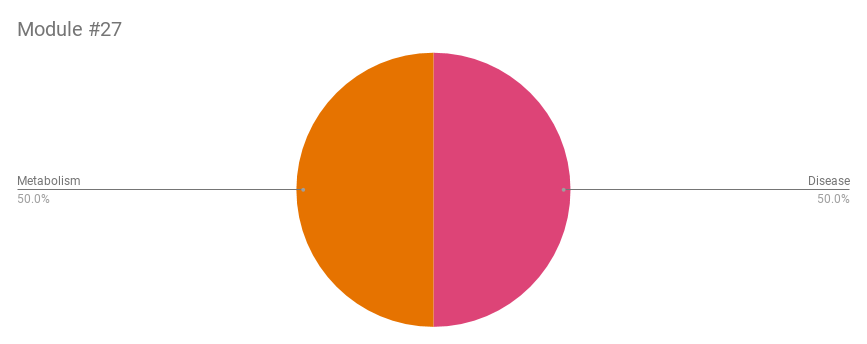
**

**
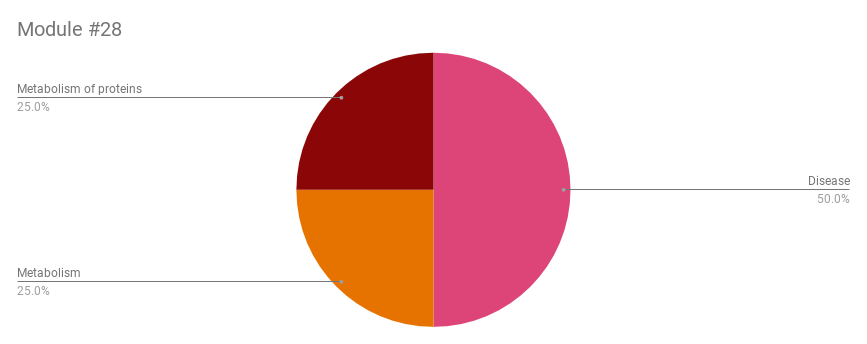
**

**
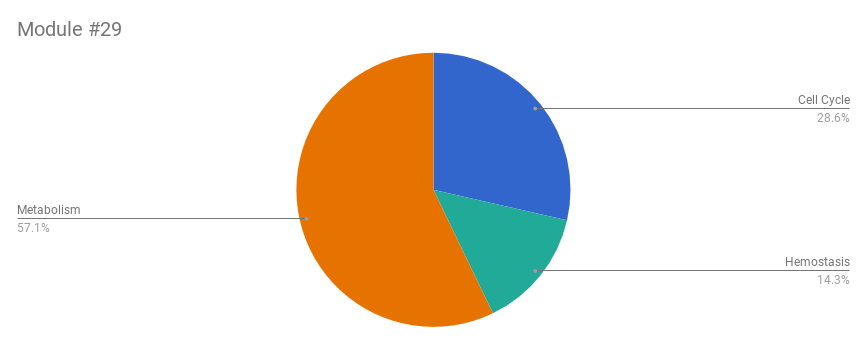
**

**
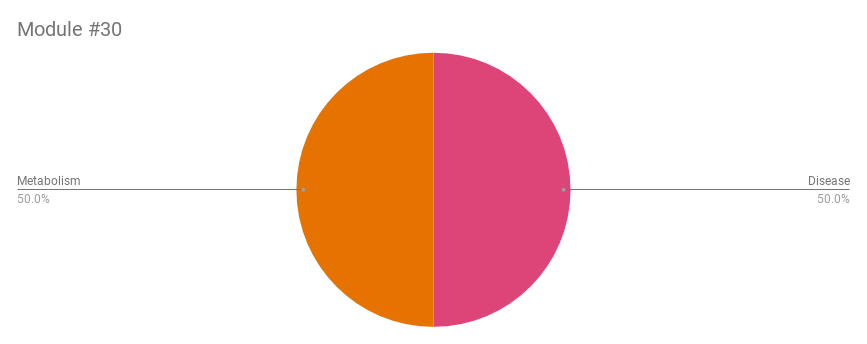
**

**
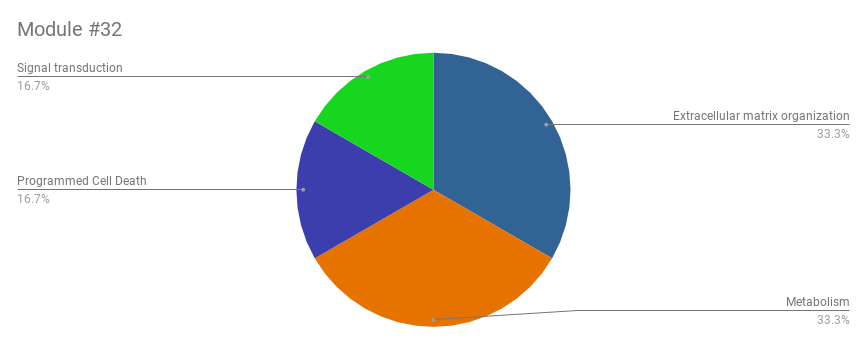
**

**
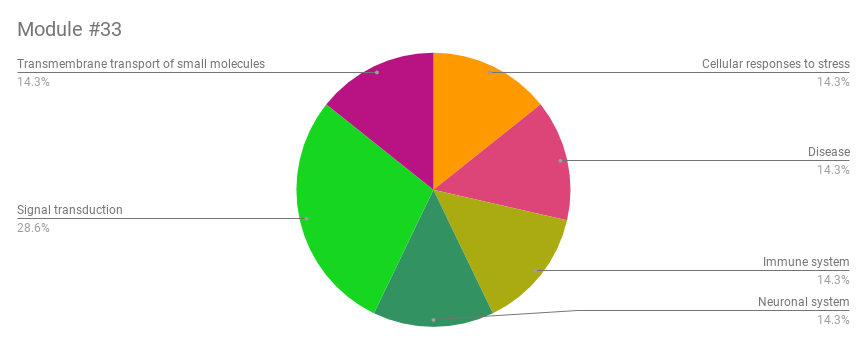
**

**
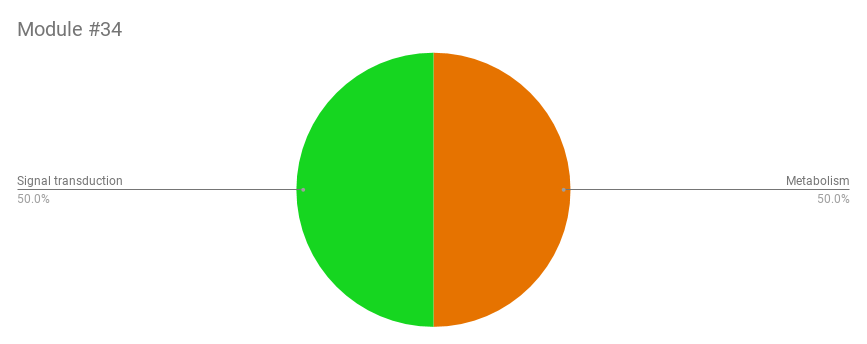
**

**
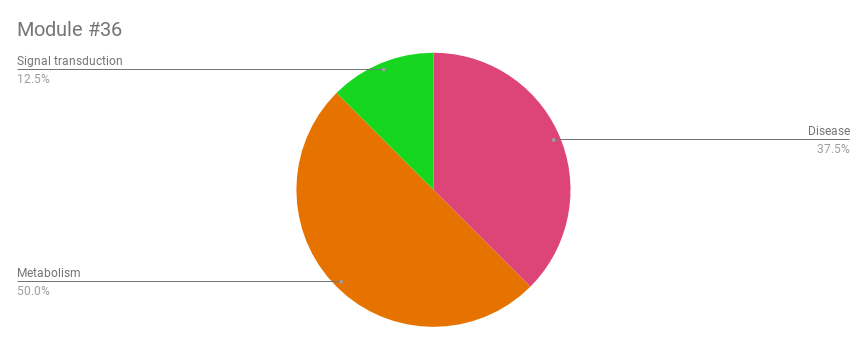
**

**
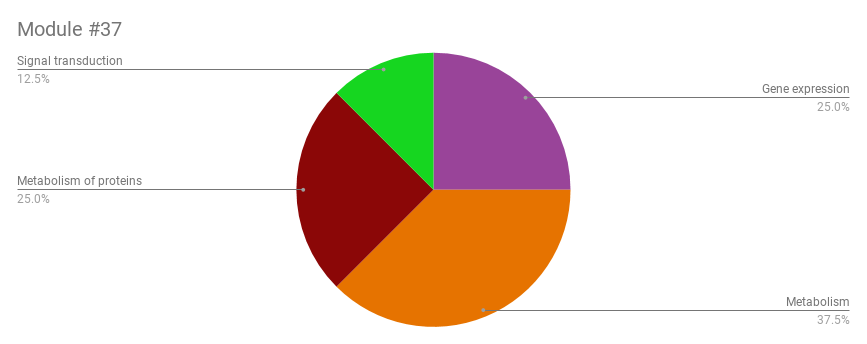
**

**
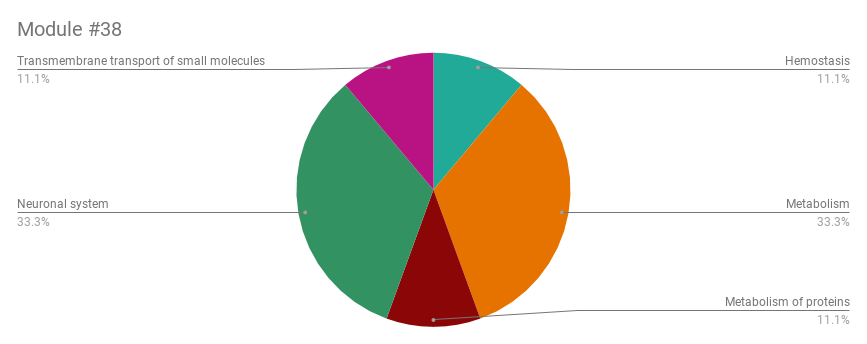
**

**
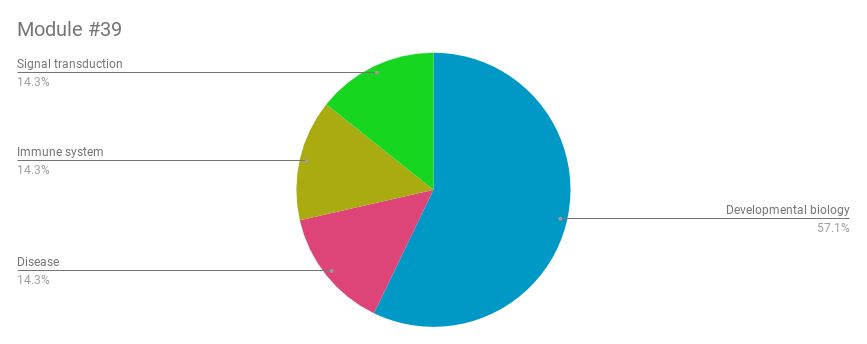
**

**
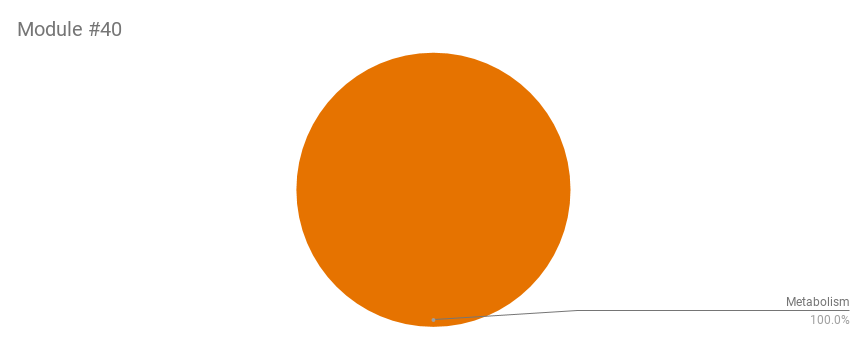
**

**Probes list for each module:**

Module #1:

A_23_P101642

A_23_P105794

A_23_P108028

A_23_P111672

A_23_P112673

A_23_P114649

A_23_P116123

A_23_P116890

A_23_P120227

A_23_P120572

A_23_P12113

A_23_P121527

A_23_P122052

A_23_P12816

A_23_P129301

A_23_P131846

A_23_P133902

A_23_P134744

A_23_P136805

A_23_P136817

A_23_P138099

A_23_P141345

A_23_P141376

A_23_P141730

A_23_P144697

A_23_P145916

A_23_P147166

A_23_P148475

A_23_P148807

A_23_P149111

A_23_P14957

A_23_P152218

A_23_P153945

A_23_P156310

A_23_P160537

A_23_P164081

A_23_P166297

A_23_P167276

A_23_P168443

A_23_P168651

A_23_P18102

A_23_P200425

A_23_P201979

A_23_P202280

A_23_P202361

A_23_P202392

A_23_P202773

A_23_P204581

A_23_P204998

A_23_P205746

A_23_P205789

A_23_P206454

A_23_P206612

A_23_P207456

A_23_P210425

A_23_P214908

A_23_P215599

A_23_P216610

A_23_P216920

A_23_P218827

A_23_P23303

A_23_P24244

A_23_P250118

A_23_P251104

A_23_P25194

A_23_P252211

A_23_P252740

A_23_P253752

A_23_P254612

A_23_P25626

A_23_P256473

A_23_P256956

A_23_P259586

A_23_P29005

A_23_P300150

A_23_P302005

A_23_P310956

A_23_P311616

A_23_P312174

A_23_P31224

A_23_P314526

A_23_P314712

A_23_P317056

A_23_P319617

A_23_P321261

A_23_P327307

A_23_P328740

A_23_P333951

A_23_P339053

A_23_P347040

A_23_P34757

A_23_P352799

A_23_P359854

A_23_P360605

A_23_P36140

A_23_P361448

A_23_P363968

A_23_P364504

A_23_P366253

A_23_P368711

A_23_P371284

A_23_P37311

A_23_P377957

A_23_P379864

A_23_P383060

A_23_P389391

A_23_P393713

A_23_P397055

A_23_P39925

A_23_P401547

A_23_P405761

A_23_P408271

A_23_P408830

A_23_P410312

A_23_P414978

A_23_P416036

A_23_P429491

A_23_P43296

A_23_P4536

A_23_P46095

A_23_P46337

A_23_P47155

A_23_P48029

A_23_P502336

A_23_P502641

A_23_P52634

A_23_P53458

A_23_P54313

A_23_P55518

A_23_P57199

A_23_P57588

A_23_P58009

A_23_P58132

A_23_P59138

A_23_P65983

A_23_P67339

A_23_P71067

A_23_P71415

A_23_P71570

A_23_P74914

A_23_P75310

A_23_P79622

A_23_P79692

A_23_P80032

A_23_P82108

A_23_P8241

A_23_P82738

A_23_P83498

A_23_P83714

A_23_P88630

A_23_P92057

A_23_P92860

A_23_P93737

A_23_P97021

A_24_P120303

A_24_P123347

A_24_P12397

A_24_P126741

A_24_P141629

A_24_P156049

A_24_P158089

A_24_P159434

A_24_P169343

A_24_P207139

A_24_P208345

A_24_P212096

A_24_P213144

A_24_P213794

A_24_P216361

A_24_P21715

A_24_P225679

A_24_P229377

A_24_P235049

A_24_P250922

A_24_P261083

A_24_P2648

A_24_P267452

A_24_P269687

A_24_P276628

A_24_P296508

A_24_P313397

A_24_P314571

A_24_P326660

A_24_P337388

A_24_P348090

A_24_P349648

A_24_P365526

A_24_P366082

A_24_P367397

A_24_P370372

A_24_P370484

A_24_P390096

A_24_P391586

A_24_P393312

A_24_P396197

A_24_P407866

A_24_P479065

A_24_P55092

A_24_P59596

A_24_P671842

A_24_P678104

A_24_P698136

A_24_P702813

A_24_P71244

A_24_P74981

A_24_P79054

A_24_P84711

A_24_P85775

A_24_P85942

A_24_P871726

A_24_P88763

A_24_P89457

A_24_P89708

A_24_P902509

A_24_P911676

A_24_P91222

A_24_P913005

A_24_P915196

A_24_P924697

A_24_P925040

A_24_P925062

A_24_P935819

A_24_P943040

A_24_P944570

A_24_P96474

A_32_P104617

A_32_P108666

A_32_P117170

A_32_P117464

A_32_P12610

A_32_P139894

A_32_P140079

A_32_P144629

A_32_P148476

A_32_P150891

A_32_P15128

A_32_P161681

A_32_P162187

A_32_P172114

A_32_P193080

A_32_P201958

A_32_P217750

A_32_P220881

A_32_P27041

A_32_P318086

A_32_P353677

A_32_P35800

A_32_P494169

A_32_P49848

A_32_P50452

A_32_P5251

A_32_P53713

A_32_P56001

A_32_P66625

A_32_P70519

A_32_P82895

A_32_P831181

A_32_P86739

A_32_P96719

Module #2:

A_23_P103981

A_23_P108751

A_23_P112482

A_23_P121702

A_23_P12514

A_23_P145264

A_23_P152876

A_23_P153745

A_23_P162866

A_23_P162874

A_23_P200507

A_23_P201459

A_23_P202520

A_23_P205031

A_23_P207520

A_23_P211212

A_23_P2492

A_23_P25735

A_23_P2661

A_23_P31315

A_23_P32404

A_23_P334709

A_23_P37205

A_23_P373126

A_23_P394064

A_23_P399501

A_23_P408353

A_23_P45524

A_23_P45917

A_23_P49338

A_23_P500000

A_23_P50498

A_23_P50504

A_23_P59005

A_23_P62659

A_23_P66347

A_23_P69179

A_23_P7642

A_23_P78265

A_23_P91802

A_24_P116351

A_24_P145377

A_24_P15502

A_24_P254933

A_24_P277934

A_24_P376483

A_24_P7040

A_24_P783679

A_24_P80204

A_24_P868905

A_24_P912382

A_32_P151544

A_32_P155247

A_32_P163469

A_32_P218707

A_32_P32254

Module #3

A_23_P102202

A_23_P108662

A_23_P109201

A_23_P110837

A_23_P111000

A_23_P113212

A_23_P123974

A_23_P125265

A_23_P127426

A_23_P130194

A_23_P131935

A_23_P132784

A_23_P134454

A_23_P137366

A_23_P138725

A_23_P145238

A_23_P153026

A_23_P157072

A_23_P160559

A_23_P161698

A_23_P162047

A_23_P165598

A_23_P168868

A_23_P17870

A_23_P203900

A_23_P208674

A_23_P210176

A_23_P212617

A_23_P2271

A_23_P24716

A_23_P24870

A_23_P254340

A_23_P254512

A_23_P255111

A_23_P256455

A_23_P257649

A_23_P27994

A_23_P29551

A_23_P29773

A_23_P29803

A_23_P338113

A_23_P34744

A_23_P376211

A_23_P39465

A_23_P40174

A_23_P411296

A_23_P41280

A_23_P434352

A_23_P49155

A_23_P50096

A_23_P502425

A_23_P52266

A_23_P55706

A_23_P57667

A_23_P65651

A_23_P6708

A_23_P6935

A_23_P7313

A_23_P73429

A_23_P75509

A_23_P78664

A_23_P80068

A_23_P82169

A_23_P82299

A_23_P87216

A_23_P88119

A_23_P89780

A_23_P91081

A_23_P91590

A_23_P94533

A_23_P97990

A_23_P98304

A_23_P99063

A_24_P101402

A_24_P12626

A_24_P180680

A_24_P184555

A_24_P219552

A_24_P237878

A_24_P263767

A_24_P269527

A_24_P341126

A_24_P365807

A_24_P48898

A_24_P68631

A_24_P8371

A_24_P87931

A_32_P147241

A_32_P171328

A_32_P192430

A_32_P94444

Module #4

A_23_P100486

A_23_P100711

A_23_P104651

A_23_P110504

A_23_P111041

A_23_P114783

A_23_P115313

A_23_P118246

A_23_P120316

A_23_P122197

A_23_P122863

A_23_P124022

A_23_P124084

A_23_P12911

A_23_P131706

A_23_P134314

A_23_P135634

A_23_P14184

A_23_P142750

A_23_P143916

A_23_P150249

A_23_P152655

A_23_P155027

A_23_P155316

A_23_P155624

A_23_P15582

A_23_P157371

A_23_P159927

A_23_P160849

A_23_P163506

A_23_P165657

A_23_P168229

A_23_P170058

A_23_P18196

A_23_P18604

A_23_P19712

A_23_P200443

A_23_P203299

A_23_P203332

A_23_P204937

A_23_P20502

A_23_P208551

A_23_P211910

A_23_P215566

A_23_P215790

A_23_P24997

A_23_P250042

A_23_P252928

A_23_P254573

A_23_P2554

A_23_P258272

A_23_P259442

A_23_P259506

A_23_P28886

A_23_P30805

A_23_P310317

A_23_P32253

A_23_P331479

A_23_P331928

A_23_P332992

A_23_P34788

A_23_P358944

A_23_P360874

A_23_P363275

A_23_P36364

A_23_P366216

A_23_P37484

A_23_P374844

A_23_P37704

A_23_P379736

A_23_P3866

A_23_P387057

A_23_P391778

A_23_P398460

A_23_P404494

A_23_P40896

A_23_P414273

A_23_P423864

A_23_P428129

A_23_P46470

A_23_P50108

A_23_P50477

A_23_P51397

A_23_P52207

A_23_P52298

A_23_P53276

A_23_P57379

A_23_P57697

A_23_P5983

A_23_P68155

A_23_P69437

A_23_P69908

A_23_P70398

A_23_P7423

A_23_P75811

A_23_P77493

A_23_P90612

A_23_P92261

A_23_P95764

A_23_P99927

A_24_P107695

A_24_P107941

A_24_P118142

A_24_P128880

A_24_P130296

A_24_P149124

A_24_P165259

A_24_P17031

A_24_P18190

A_24_P183094

A_24_P191067

A_24_P218265

A_24_P223124

A_24_P271527

A_24_P278637

A_24_P297539

A_24_P305764

A_24_P307126

A_24_P309317

A_24_P313744

A_24_P32715

A_24_P335620

A_24_P340066

A_24_P398781

A_24_P402690

A_24_P416131

A_24_P418408

A_24_P462899

A_24_P823684

A_24_P921366

A_24_P931443

A_32_P114574

A_32_P162183

A_32_P215938

A_32_P28365

A_32_P49284

A_32_P49844

A_32_P62863

A_32_P90047

A_32_P99100

Module #5

A_23_P100292

A_23_P106859

A_23_P106906

A_23_P107401

A_23_P110167

A_23_P112251

A_23_P114057

A_23_P115091

A_23_P115482

A_23_P117852

A_23_P118815

A_23_P125624

A_23_P132874

A_23_P134295

A_23_P135494

A_23_P13663

A_23_P141688

A_23_P145694

A_23_P146367

A_23_P151267

A_23_P160968

A_23_P166306

A_23_P166899

A_23_P169629

A_23_P18372

A_23_P19226

A_23_P19987

A_23_P201551

A_23_P20196

A_23_P203406

A_23_P206059

A_23_P206103

A_23_P206510

A_23_P206901

A_23_P208779

A_23_P21033

A_23_P213678

A_23_P216630

A_23_P217866

A_23_P218523

A_23_P24345

A_23_P251771

A_23_P251893

A_23_P254702

A_23_P303087

A_23_P30363

A_23_P31399

A_23_P326160

A_23_P359245

A_23_P360316

A_23_P366366

A_23_P372962

A_23_P382

A_23_P39074

A_23_P401

A_23_P406424

A_23_P42335

A_23_P430068

A_23_P434890

A_23_P50907

A_23_P51906

A_23_P57118

A_23_P59210

A_23_P65757

A_23_P6624

A_23_P66355

A_23_P67864

A_23_P69249

A_23_P69537

A_23_P71727

A_23_P73763

A_23_P74034

A_23_P7873

A_23_P85441

A_23_P8763

A_23_P91328

A_23_P93750

A_23_P9574

A_23_P9756

A_24_P104091

A_24_P105102

A_24_P114604

A_24_P13790

A_24_P149645

A_24_P150361

A_24_P187948

A_24_P195714

A_24_P226008

A_24_P240166

A_24_P269315

A_24_P284893

A_24_P303524

A_24_P304051

A_24_P313504

A_24_P362317

A_24_P3783

A_24_P379750

A_24_P408424

A_24_P55295

A_24_P673968

A_24_P693986

A_24_P736638

A_24_P876862

A_32_P184933

A_32_P197489

A_32_P199252

A_32_P199725

A_32_P218355

A_32_P72447

A_32_P75122

Module #6

A_23_P111995

A_23_P112026

A_23_P131614

A_23_P133656

A_23_P14062

A_23_P150979

A_23_P213050

A_23_P215060

A_23_P348257

A_23_P42718

A_23_P52697

A_23_P61987

A_23_P65240

A_23_P74290

A_23_P76291

A_23_P80040

A_23_P89431

A_24_P248251

A_24_P298805

A_24_P404822

A_24_P935491

A_32_P70158

Module #7

A_23_P109143

A_23_P127584

A_23_P131676

A_23_P132956

A_23_P142075

A_23_P148255

A_23_P150609

A_23_P158148

A_23_P17053

A_23_P202964

A_23_P207850

A_23_P213385

A_23_P215634

A_23_P218675

A_23_P24444

A_23_P258321

A_23_P340698

A_23_P428184

A_23_P71241

A_23_P82868

A_23_P88522

A_23_P98350

A_24_P115762

A_32_P54544

Module #8

A_23_P110204

A_23_P121064

A_23_P134835

A_23_P146347

A_23_P164451

A_23_P19182

A_23_P203947

A_23_P205419

A_23_P206212

A_23_P212665

A_23_P212844

A_23_P217564

A_23_P217968

A_23_P28815

A_23_P2960

A_23_P301304

A_23_P345065

A_23_P39517

A_23_P426944

A_23_P82651

A_23_P87879

A_23_P90696

A_23_P91970

A_23_P93722

A_23_P96369

A_24_P110983

A_24_P122137

A_24_P192485

A_24_P247074

A_24_P347065

A_24_P409115

A_24_P471242

A_24_P838448

A_24_P93111

A_32_P165477

A_32_P170736

A_32_P184268

A_32_P212886

A_32_P228438

A_32_P72351

Module #9

A_23_P103775

A_23_P10605

A_23_P106798

A_23_P10858

A_23_P109304

A_23_P115478

A_23_P118174

A_23_P122439

A_23_P124384

A_23_P127288

A_23_P12767

A_23_P128613

A_23_P128808

A_23_P1331

A_23_P137423

A_23_P14458

A_23_P150667

A_23_P151415

A_23_P151856

A_23_P152620

A_23_P161474

A_23_P163099

A_23_P165408

A_23_P201676

A_23_P205778

A_23_P206077

A_23_P206441

A_23_P207400

A_23_P20876

A_23_P209135

A_23_P209669

A_23_P213137

A_23_P216396

A_23_P23522

A_23_P24260

A_23_P251686

A_23_P251795

A_23_P256033

A_23_P256297

A_23_P29083

A_23_P300728

A_23_P301877

A_23_P304237

A_23_P310590

A_23_P31177

A_23_P320242

A_23_P32064

A_23_P322519

A_23_P327519

A_23_P330486

A_23_P343843

A_23_P348138

A_23_P35219

A_23_P355075

A_23_P356041

A_23_P361419

A_23_P36865

A_23_P371215

A_23_P39647

A_23_P397285

A_23_P40718

A_23_P41948

A_23_P425502

A_23_P429560

A_23_P430120

A_23_P43248

A_23_P43786

A_23_P46539

A_23_P48951

A_23_P52278

A_23_P59700

A_23_P60488

A_23_P65555

A_23_P69310

A_23_P69452

A_23_P69738

A_23_P70677

A_23_P70794

A_23_P74663

A_23_P75973

A_23_P79259

A_23_P80940

A_23_P81811

A_23_P82394

A_23_P82979

A_23_P89601

A_23_P90497

A_23_P94053

A_24_P100234

A_24_P11436

A_24_P11462

A_24_P122337

A_24_P129417

A_24_P133162

A_24_P134372

A_24_P14156

A_24_P17677

A_24_P17710

A_24_P17719

A_24_P177631

A_24_P195400

A_24_P196665

A_24_P201739

A_24_P216654

A_24_P223518

A_24_P23034

A_24_P234094

A_24_P23522

A_24_P238499

A_24_P238578

A_24_P23865

A_24_P255123

A_24_P257099

A_24_P258051

A_24_P261125

A_24_P269779

A_24_P278156

A_24_P283000

A_24_P287075

A_24_P288722

A_24_P29733

A_24_P300841

A_24_P305345

A_24_P305556

A_24_P312119

A_24_P317637

A_24_P336728

A_24_P343095

A_24_P348806

A_24_P354300

A_24_P358462

A_24_P35935

A_24_P392496

A_24_P394698

A_24_P397107

A_24_P42603

A_24_P43876

A_24_P476718

A_24_P479510

A_24_P5550

A_24_P56240

A_24_P567408

A_24_P635355

A_24_P67494

A_24_P68585

A_24_P7143

A_24_P73962

A_24_P75408

A_24_P77904

A_24_P832426

A_24_P83678

A_24_P84608

A_24_P876408

A_24_P901856

A_24_P912856

A_24_P916195

A_24_P918436

A_24_P919727

A_24_P942481

A_24_P94351

A_24_P96780

A_32_P119165

A_32_P119744

A_32_P120484

A_32_P134679

A_32_P136427

A_32_P139654

A_32_P147969

A_32_P149735

A_32_P151102

A_32_P151800

A_32_P15464

A_32_P169179

A_32_P173439

A_32_P190049

A_32_P199292

A_32_P204205

A_32_P217901

A_32_P22191

A_32_P38003

A_32_P406142

A_32_P50209

A_32_P64475

A_32_P6917

A_32_P69803

A_32_P75661

A_32_P82475

A_32_P96134

Module #10

A_23_P104193

A_23_P110531

A_23_P117104

A_23_P122924

A_23_P125423

A_23_P13094

A_23_P139704

A_23_P150316

A_23_P151506

A_23_P164650

A_23_P165927

A_23_P168788

A_23_P18452

A_23_P200728

A_23_P203475

A_23_P212696

A_23_P214168

A_23_P216501

A_23_P218770

A_23_P218774

A_23_P25121

A_23_P33196

A_23_P33723

A_23_P365719

A_23_P370707

A_23_P373017

A_23_P391586

A_23_P395438

A_23_P57089

A_23_P69720

A_23_P76480

A_23_P77612

A_23_P88865

A_23_P8913

A_23_P94030

A_24_P141214

A_24_P166663

A_24_P236799

A_24_P28722

A_24_P335092

A_24_P413126

A_24_P598836

A_24_P9671

A_32_P217655

A_32_P51988

A_32_P74409

Module #11

A_23_P105392

A_23_P106002

A_23_P107421

A_23_P112481

A_23_P115955

A_23_P119337

A_23_P120364

A_23_P124619

A_23_P125107

A_23_P125109

A_23_P134176

A_23_P142533

A_23_P145904

A_23_P147918

A_23_P151975

A_23_P156327

A_23_P163782

A_23_P165840

A_23_P166459

A_23_P1691

A_23_P200866

A_23_P201636

A_23_P214330

A_23_P24104

A_23_P252642

A_23_P254507

A_23_P257672

A_23_P257863

A_23_P2601

A_23_P314024

A_23_P350295

A_23_P353035

A_23_P37441

A_23_P37983

A_23_P39364

A_23_P41114

A_23_P417404

A_23_P427703

A_23_P53476

A_23_P60933

A_23_P61688

A_23_P68106

A_23_P7144

A_23_P72737

A_23_P81898

A_23_P819

A_23_P85250

A_23_P86917

A_23_P87545

A_23_P93690

A_23_P95917

A_24_P16124

A_24_P161933

A_24_P199905

A_24_P270460

A_24_P287043

A_24_P418044

A_24_P860781

A_32_P154256

A_32_P167592

A_32_P25273

A_32_P34138

A_32_P95397

Module #12

A_23_P103951

A_23_P106024

A_23_P107465

A_23_P11372

A_23_P118266

A_23_P120002

A_23_P125278

A_23_P130995

A_23_P135239

A_23_P136870

A_23_P138931

A_23_P150693

A_23_P152782

A_23_P155755

A_23_P156049

A_23_P162171

A_23_P16469

A_23_P167096

A_23_P206018

A_23_P20814

A_23_P21644

A_23_P218131

A_23_P253052

A_23_P308763

A_23_P321501

A_23_P321703

A_23_P321920

A_23_P35021

A_23_P35412

A_23_P360754

A_23_P364024

A_23_P37685

A_23_P384024

A_23_P38795

A_23_P406616

A_23_P4283

A_23_P45365

A_23_P45475

A_23_P45871

A_23_P47955

A_23_P48570

A_23_P487

A_23_P56567

A_23_P59261

A_23_P6413

A_23_P64792

A_23_P70660

A_23_P75786

A_23_P76006

A_23_P76109

A_23_P80098

A_23_P80839

A_23_P82449

A_23_P8513

A_24_P117410

A_24_P165949

A_24_P194081

A_24_P195164

A_24_P20607

A_24_P222655

A_24_P228130

A_24_P251764

A_24_P261417

A_24_P262201

A_24_P319923

A_24_P323298

A_24_P355816

A_24_P414999

A_24_P56388

A_24_P567298

A_24_P696761

A_24_P84428

A_24_P924484

A_24_P95154

A_32_P107372

A_32_P112546

A_32_P175098

A_32_P399546

Module #13

A_23_P107981

A_23_P11025

A_23_P123071

A_23_P137173

A_23_P143906

A_23_P152548

A_23_P158096

A_23_P169137

A_23_P18751

A_23_P210482

A_23_P2423

A_23_P24843

A_23_P2920

A_23_P314151

A_23_P360777

A_23_P432978

A_23_P500421

A_23_P54968

A_23_P62807

A_23_P69497

A_23_P7827

A_23_P8834

A_23_P92562

A_23_P95930

A_24_P109101

A_24_P235266

A_24_P299685

A_24_P301146

A_24_P367432

A_24_P409881

A_24_P83262

A_24_P870620

A_24_P95070

A_32_P132206

A_32_P161855

A_32_P25889

A_32_P69149

Module #14

A_23_P101407

A_23_P114883

A_23_P114947

A_23_P160318

A_23_P166408

A_23_P170733

A_23_P203540

A_23_P21882

A_23_P23443

A_23_P254654

A_23_P30813

A_23_P31453

A_23_P315364

A_23_P32444

A_23_P353717

A_23_P35414

A_23_P43164

A_23_P52761

A_23_P56898

A_23_P59268

A_23_P59950

A_23_P74609

A_23_P94380

A_24_P257416

A_24_P277367

A_24_P489399

A_24_P703642

Module #15

A_23_P102731

A_23_P10391

A_23_P104563

A_23_P106532

A_23_P106773

A_23_P107775

A_23_P110403

A_23_P111888

A_23_P113056

A_23_P117782

A_23_P118065

A_23_P119202

A_23_P123234

A_23_P123265

A_23_P136978

A_23_P137381

A_23_P138567

A_23_P139104

A_23_P141191

A_23_P144244

A_23_P144916

A_23_P145657

A_23_P147224

A_23_P15174

A_23_P153964

A_23_P156025

A_23_P156687

A_23_P157299

A_23_P157879

A_23_P158593

A_23_P158925

A_23_P163467

A_23_P18078

A_23_P19333

A_23_P204541

A_23_P205057

A_23_P207058

A_23_P211233

A_23_P214144

A_23_P215549

A_23_P216340

A_23_P218111

A_23_P23346

A_23_P25069

A_23_P252721

A_23_P257144

A_23_P259621

A_23_P320578

A_23_P3312

A_23_P3532

A_23_P372946

A_23_P383986

A_23_P391344

A_23_P40108

A_23_P410613

A_23_P413641

A_23_P41424

A_23_P414913

A_23_P41734

A_23_P420551

A_23_P421401

A_23_P429977

A_23_P49878

A_23_P502520

A_23_P50426

A_23_P50919

A_23_P56746

A_23_P58396

A_23_P60079

A_23_P74278

A_23_P87011

A_23_P92499

A_23_P99163

A_24_P11506

A_24_P298179

A_24_P316059

A_24_P348326

A_24_P788772

A_24_P85539

A_24_P98411

A_32_P142440

A_32_P175301

A_32_P192842

A_32_P29703

A_32_P89709

Module #16

A_23_P103672

A_23_P103765

A_23_P113393

A_23_P116037

A_23_P118894

A_23_P121596

A_23_P124108

A_23_P143981

A_23_P146512

A_23_P148249

A_23_P150018

A_23_P163087

A_23_P165848

A_23_P166823

A_23_P207911

A_23_P210100

A_23_P216429

A_23_P218928

A_23_P251499

A_23_P27606

A_23_P32707

A_23_P32793

A_23_P33326

A_23_P34345

A_23_P376488

A_23_P396765

A_23_P413150

A_23_P414793

A_23_P416581

A_23_P422831

A_23_P433016

A_23_P5131

A_23_P57417

A_23_P63736

A_23_P65678

A_23_P66682

A_23_P67169

A_23_P80048

A_23_P8083

A_23_P83818

A_23_P87013

A_23_P87752

A_24_P131522

A_24_P166613

A_24_P181055

A_24_P244800

A_24_P261032

A_24_P267523

A_24_P282383

A_24_P316127

A_24_P357950

A_24_P511686

A_24_P517918

A_24_P595223

A_24_P605612

A_24_P608268

A_24_P658584

A_24_P79403

A_32_P127978

A_32_P153361

A_32_P173662

A_32_P47754

Module #17

A_23_P100344

A_23_P101048

A_23_P110052

A_23_P128974

A_23_P130182

A_23_P14432

A_23_P144896

A_23_P147822

A_23_P156289

A_23_P163475

A_23_P165624

A_23_P1936

A_23_P205828

A_23_P213562

A_23_P343411

A_23_P354170

A_23_P36562

A_23_P3681

A_23_P38723

A_23_P389919

A_23_P412554

A_23_P51231

A_23_P51767

A_23_P52480

A_23_P5392

A_23_P54918

A_23_P5611

A_23_P56978

A_23_P57227

A_23_P63010

A_23_P68072

A_23_P73972

A_23_P77328

A_24_P10137

A_24_P104512

A_24_P161525

A_24_P236091

A_24_P24685

A_24_P270033

A_24_P273014

A_24_P324314

A_24_P346431

A_24_P347310

A_24_P36425

A_24_P390060

A_24_P535256

A_24_P918317

A_32_P116556

A_32_P122703

A_32_P138178

A_32_P156531

A_32_P300427

A_32_P32406

A_32_P50066

Module #18

A_23_P101759

A_23_P10194

A_23_P108604

A_23_P109420

A_23_P110122

A_23_P111126

A_23_P114952

A_23_P115872

A_23_P117599

A_23_P118392

A_23_P119102

A_23_P120467

A_23_P12292

A_23_P13183

A_23_P132365

A_23_P138507

A_23_P139418

A_23_P144071

A_23_P146798

A_23_P147431

A_23_P154566

A_23_P157865

A_23_P160546

A_23_P166360

A_23_P166508

A_23_P201672

A_23_P205188

A_23_P205370

A_23_P207213

A_23_P208450

A_23_P210726

A_23_P215658

A_23_P218086

A_23_P218751

A_23_P250122

A_23_P252371

A_23_P254816

A_23_P258862

A_23_P27584

A_23_P28485

A_23_P29594

A_23_P30655

A_23_P31584

A_23_P335329

A_23_P336670

A_23_P345139

A_23_P35114

A_23_P361049

A_23_P38346

A_23_P386

A_23_P39076

A_23_P392384

A_23_P394836

A_23_P408094

A_23_P411833

A_23_P42375

A_23_P4286

A_23_P434118

A_23_P45999

A_23_P500130

A_23_P502274

A_23_P502312

A_23_P50455

A_23_P52017

A_23_P53247

A_23_P54291

A_23_P63789

A_23_P63980

A_23_P65110

A_23_P69383

A_23_P69586

A_23_P73593

A_23_P75741

A_23_P79978

A_23_P82478

A_23_P873

A_23_P87560

A_23_P88740

A_23_P89509

A_23_P897

A_23_P95302

A_23_P98261

A_23_P99771

A_24_P135276

A_24_P156113

A_24_P164388

A_24_P170295

A_24_P192994

A_24_P21044

A_24_P213494

A_24_P225604

A_24_P255314

A_24_P255954

A_24_P262395

A_24_P274270

A_24_P277295

A_24_P279760

A_24_P285623

A_24_P324506

A_24_P337796

A_24_P339201

A_24_P355944

A_24_P372625

A_24_P388528

A_24_P38865

A_24_P38951

A_24_P405205

A_24_P46171

A_24_P506977

A_24_P55437

A_24_P567454

A_24_P586712

A_24_P59667

A_24_P63262

A_24_P67681

A_24_P684799

A_24_P690983

A_24_P761727

A_24_P76644

A_24_P80633

A_24_P98086

A_32_P103291

A_32_P114284

A_32_P116813

A_32_P143824

A_32_P149416

A_32_P194848

A_32_P202708

A_32_P206698

A_32_P25050

A_32_P32653

A_32_P44274

A_32_P514599

A_32_P52609

A_32_P90695

Module #19

A_23_P111531

A_23_P121480

A_23_P144959

A_23_P159382

A_23_P217236

A_23_P218646

A_23_P24784

A_23_P256142

A_23_P257372

A_23_P34307

A_23_P344421

A_23_P360626

A_23_P389897

A_23_P60259

A_23_P60856

A_23_P73848

A_23_P75516

A_23_P79482

A_23_P83134

A_24_P157698

A_24_P187954

A_24_P24053

A_24_P409661

A_24_P940576

A_32_P117354

A_32_P230720

A_32_P49035

A_32_P75284

Module #20

A_23_P10182

A_23_P115608

A_23_P13083

A_23_P131990

A_23_P13679

A_23_P147665

A_23_P152995

A_23_P19102

A_23_P200999

A_23_P203629

A_23_P205500

A_23_P209731

A_23_P215744

A_23_P217168

A_23_P23457

A_23_P250951

A_23_P258912

A_23_P25913

A_23_P309246

A_23_P335069

A_23_P355517

A_23_P399851

A_23_P401472

A_23_P4069

A_23_P409723

A_23_P46025

A_23_P46063

A_23_P47980

A_23_P52657

A_23_P66637

A_23_P82550

A_23_P89302

A_23_P93940

A_23_P94133

A_23_P96271

A_24_P108301

A_24_P131622

A_24_P186370

A_24_P277747

A_24_P315256

A_24_P319942

A_24_P320796

A_24_P329353

A_24_P331150

A_24_P341909

A_24_P367079

A_24_P379413

A_24_P383523

A_24_P398130

A_24_P40626

A_24_P45476

A_24_P52887

A_24_P56363

A_24_P62800

A_24_P706752

A_24_P80532

A_24_P88031

A_24_P90349

A_24_P914000

A_24_P921321

A_24_P942163

A_32_P105397

A_32_P129573

A_32_P131031

A_32_P171903

A_32_P184888

A_32_P191285

A_32_P194062

A_32_P38989

A_32_P7015

A_32_P83098

Module #21

A_23_P101246

A_23_P111766

A_23_P135257

A_23_P139786

A_23_P150789

A_23_P152838

A_23_P160167

A_23_P16523

A_23_P23074

A_23_P24004

A_23_P26954

A_23_P306203

A_23_P310274

A_23_P350396

A_23_P50269

A_23_P71037

A_23_P76743

A_24_P101651

A_24_P107277

A_24_P11061

A_24_P117620

A_24_P14464

A_24_P237586

A_24_P273647

A_24_P303091

A_24_P402242

A_24_P414269

A_24_P686965

A_24_P79529

A_32_P44394

A_32_P4882

Module #22

A_23_P110764

A_23_P148990

A_23_P154818

A_23_P20316

A_23_P309701

A_23_P343366

A_23_P374310

A_23_P41128

A_23_P49657

A_23_P51787

A_23_P61498

A_23_P72462

A_23_P76622

A_24_P119201

A_24_P123408

A_24_P179044

A_24_P229871

A_24_P269062

A_24_P272310

A_24_P308079

A_24_P316904

A_24_P317907

A_24_P395610

A_24_P407259

A_24_P481783

A_24_P595460

A_24_P778844

A_24_P799680

A_24_P90005

A_24_P924543

A_24_P940327

A_24_P94319

A_32_P150086

A_32_P157124

A_32_P167017

A_32_P170905

A_32_P229618

A_32_P73452

A_32_P775081

Module #23

A_23_P102192

A_23_P102412

A_23_P102508

A_23_P102551

A_23_P102769

A_23_P10385

A_23_P104073

A_23_P10701

A_23_P108871

A_23_P113825

A_23_P114275

A_23_P117654

A_23_P119254

A_23_P119266

A_23_P120056

A_23_P12282

A_23_P124417

A_23_P125977

A_23_P133123

A_23_P136232

A_23_P138426

A_23_P138465

A_23_P138495

A_23_P139123

A_23_P143068

A_23_P143120

A_23_P143147

A_23_P143190

A_23_P146584

A_23_P149042

A_23_P155057

A_23_P158596

A_23_P158794

A_23_P159390

A_23_P1602

A_23_P168532

A_23_P170491

A_23_P170667

A_23_P19352

A_23_P19455

A_23_P200222

A_23_P203888

A_23_P204087

A_23_P204980

A_23_P205997

A_23_P206107

A_23_P209167

A_23_P209678

A_23_P210253

A_23_P211428

A_23_P211748

A_23_P212715

A_23_P215461

A_23_P216043

A_23_P216068

A_23_P217135

A_23_P22096

A_23_P24723

A_23_P250156

A_23_P25019

A_23_P250196

A_23_P253524

A_23_P253571

A_23_P255016

A_23_P255215

A_23_P256334

A_23_P25873

A_23_P26375

A_23_P27677

A_23_P28953

A_23_P30547

A_23_P313632

A_23_P31866

A_23_P321201

A_23_P32165

A_23_P329890

A_23_P330908

A_23_P331895

A_23_P333735

A_23_P336992

A_23_P337767

A_23_P340722

A_23_P350754

A_23_P356684

A_23_P37535

A_23_P384499

A_23_P385034

A_23_P406187

A_23_P406957

A_23_P40817

A_23_P413193

A_23_P416468

A_23_P424513

A_23_P425880

A_23_P429184

A_23_P43679

A_23_P44139

A_23_P44505

A_23_P44684

A_23_P45699

A_23_P47867

A_23_P49924

A_23_P502142

A_23_P50349

A_23_P50389

A_23_P56865

A_23_P57393

A_23_P57497

A_23_P57760

A_23_P57989

A_23_P58321

A_23_P60180

A_23_P64611

A_23_P67829

A_23_P72050

A_23_P73000

A_23_P75978

A_23_P76761

A_23_P78302

A_23_P79221

A_23_P79628

A_23_P8055

A_23_P86610

A_23_P88331

A_23_P8906

A_23_P92154

A_23_P94552

A_23_P9465

A_23_P96350

A_23_P96761

A_23_P96812

A_23_P99172

A_24_P102821

A_24_P103686

A_24_P114124

A_24_P12413

A_24_P127462

A_24_P147263

A_24_P148717

A_24_P151920

A_24_P153734

A_24_P153800

A_24_P161827

A_24_P176374

A_24_P180165

A_24_P191664

A_24_P198598

A_24_P227831

A_24_P235429

A_24_P236522

A_24_P240732

A_24_P247616

A_24_P284523

A_24_P297078

A_24_P303480

A_24_P320284

A_24_P325015

A_24_P328668

A_24_P332081

A_24_P335358

A_24_P337592

A_24_P37441

A_24_P379820

A_24_P385739

A_24_P399888

A_24_P411121

A_24_P418294

A_24_P44462

A_24_P643041

A_24_P687302

A_24_P743802

A_24_P752362

A_24_P75748

A_24_P76142

A_24_P799580

A_24_P827096

A_24_P857404

A_24_P878419

A_24_P941824

A_24_P94222

A_24_P943843

A_24_P98109

A_32_P100365

A_32_P103955

A_32_P108544

A_32_P113472

A_32_P114447

A_32_P1173

A_32_P128399

A_32_P129660

A_32_P140262

A_32_P167176

A_32_P167239

A_32_P168464

A_32_P170749

A_32_P188921

A_32_P195291

A_32_P19840

A_32_P209791

A_32_P215113

A_32_P21848

A_32_P220109

A_32_P223173

A_32_P232035

A_32_P34920

A_32_P351936

A_32_P53633

A_32_P63086

A_32_P63162

A_32_P827528

A_32_P97496

Module #24

A_23_P104471

A_23_P10614

A_23_P11032

A_23_P112774

A_23_P114968

A_23_P116902

A_23_P121499

A_23_P12965

A_23_P133236

A_23_P133293

A_23_P133956

A_23_P136573

A_23_P136787

A_23_P145844

A_23_P145889

A_23_P146551

A_23_P154022

A_23_P155989

A_23_P162300

A_23_P162425

A_23_P16834

A_23_P202004

A_23_P2041

A_23_P20622

A_23_P208167

A_23_P214026

A_23_P22557

A_23_P25030

A_23_P254688

A_23_P258698

A_23_P302654

A_23_P303286

A_23_P311640

A_23_P313623

A_23_P321949

A_23_P336040

A_23_P346421

A_23_P353005

A_23_P386320

A_23_P394448

A_23_P397455

A_23_P402670

A_23_P40782

A_23_P409988

A_23_P41267

A_23_P48988

A_23_P502314

A_23_P51518

A_23_P56328

A_23_P64808

A_23_P65532

A_23_P65967

A_23_P70951

A_23_P73192

A_23_P75647

A_23_P81926

A_23_P84860

A_23_P85269

A_23_P88404

A_23_P89018

A_23_P91221

A_23_P91829

A_23_P94403

A_23_P95027

A_23_P99604

A_24_P117964

A_24_P135322

A_24_P13533

A_24_P166527

A_24_P170983

A_24_P171549

A_24_P199500

A_24_P204358

A_24_P217572

A_24_P222740

A_24_P226355

A_24_P230173

A_24_P252155

A_24_P252364

A_24_P253827

A_24_P254285

A_24_P272290

A_24_P32151

A_24_P322354

A_24_P324787

A_24_P336853

A_24_P339071

A_24_P365129

A_24_P380022

A_24_P409420

A_24_P459937

A_24_P48856

A_24_P638453

A_24_P645765

A_24_P707102

A_24_P743806

A_24_P7950

A_24_P85099

A_24_P919304

A_24_P924040

A_24_P932220

A_32_P135243

A_32_P151621

A_32_P178696

A_32_P223140

A_32_P228348

A_32_P4349

A_32_P44139

A_32_P462013

A_32_P52144

A_32_P54553

A_32_P59673

A_32_P94176

Module #25

A_23_P108743

A_23_P111321

A_23_P113701

A_23_P114689

A_23_P115885

A_23_P121722

A_23_P129144

A_23_P133606

A_23_P141346

A_23_P153489

A_23_P156826

A_23_P157875

A_23_P157970

A_23_P162476

A_23_P164196

A_23_P169030

A_23_P173

A_23_P18903

A_23_P202327

A_23_P204333

A_23_P21134

A_23_P213288

A_23_P214281

A_23_P214907

A_23_P21548

A_23_P216278

A_23_P216355

A_23_P217554

A_23_P218531

A_23_P23616

A_23_P24332

A_23_P253495

A_23_P256581

A_23_P311901

A_23_P351215

A_23_P35755

A_23_P361014

A_23_P363174

A_23_P376704

A_23_P415643

A_23_P416705

A_23_P435029

A_23_P56578

A_23_P64879

A_23_P66767

A_23_P71053

A_24_P143032

A_24_P156490

A_24_P163405

A_24_P213503

A_24_P221335

A_24_P224488

A_24_P24263

A_24_P260122

A_24_P285880

A_24_P313445

A_24_P364296

A_24_P365506

A_24_P378928

A_24_P385341

A_24_P398147

A_24_P407224

A_24_P409750

A_24_P412088

A_24_P418744

A_24_P497235

A_24_P532232

A_24_P64344

A_24_P731648

A_24_P937691

A_32_P101860

A_32_P121085

A_32_P135450

A_32_P137849

A_32_P175979

A_32_P180336

A_32_P206401

A_32_P210798

A_32_P216041

A_32_P24585

A_32_P370026

A_32_P491904

A_32_P6972

A_32_P86763

A_32_P88719

Module #26

A_23_P102183

A_23_P106998

A_23_P112159

A_23_P112260

A_23_P113005

A_23_P118150

A_23_P119698

A_23_P12884

A_23_P132417

A_23_P135364

A_23_P135499

A_23_P140705

A_23_P141965

A_23_P150935

A_23_P151093

A_23_P153320

A_23_P154488

A_23_P1552

A_23_P155229

A_23_P156953

A_23_P161634

A_23_P163143

A_23_P170774

A_23_P170857

A_23_P200199

A_23_P207014

A_23_P20722

A_23_P210091

A_23_P210708

A_23_P212554

A_23_P21706

A_23_P22134

A_23_P251118

A_23_P252855

A_23_P253301

A_23_P255376

A_23_P256413

A_23_P256890

A_23_P259189

A_23_P25974

A_23_P26557

A_23_P26697

A_23_P30464

A_23_P309361

A_23_P32328

A_23_P32615

A_23_P341275

A_23_P34800

A_23_P35035

A_23_P370989

A_23_P382775

A_23_P384056

A_23_P385861

A_23_P389118

A_23_P39766

A_23_P40078

A_23_P40354

A_23_P404667

A_23_P417282

A_23_P42802

A_23_P43071

A_23_P45011

A_23_P48669

A_23_P48835

A_23_P49816

A_23_P50167

A_23_P55251

A_23_P55477

A_23_P60537

A_23_P68087

A_23_P68211

A_23_P68610

A_23_P69695

A_23_P71558

A_23_P748

A_23_P75769

A_23_P7596

A_23_P83298

A_23_P88309

A_23_P91900

A_23_P95470

A_23_P96542

A_23_P97365

A_23_P99731

A_24_P127063

A_24_P137372

A_24_P137434

A_24_P156911

A_24_P157926

A_24_P169843

A_24_P202769

A_24_P20814

A_24_P227091

A_24_P247454

A_24_P25346

A_24_P261259

A_24_P281683

A_24_P289178

A_24_P298946

A_24_P310864

A_24_P349606

A_24_P373562

A_24_P382001

A_24_P383450

A_24_P383660

A_24_P383834

A_24_P394533

A_24_P406334

A_24_P410086

A_24_P418687

A_24_P51777

A_24_P52004

A_24_P54879

A_24_P647163

A_24_P67063

A_24_P776784

A_24_P803809

A_24_P80406

A_24_P856273

A_24_P873688

A_24_P916228

A_24_P92823

A_32_P103837

A_32_P104263

A_32_P109165

A_32_P140898

A_32_P157192

A_32_P163089

A_32_P198399

A_32_P46238

A_32_P74712

A_32_P76091

A_32_P92505

A_32_P9382

A_32_P95729

A_32_P96752

Module #27

A_23_P1014

A_23_P103628

A_23_P108673

A_23_P110076

A_23_P116850

A_23_P127153

A_23_P138492

A_23_P138655

A_23_P141362

A_23_P142872

A_23_P144224

A_23_P144668

A_23_P146728

A_23_P14716

A_23_P148609

A_23_P152906

A_23_P155185

A_23_P159191

A_23_P160720

A_23_P16262

A_23_P162918

A_23_P164958

A_23_P166135

A_23_P167328

A_23_P17837

A_23_P204252

A_23_P204364

A_23_P205449

A_23_P209636

A_23_P211504

A_23_P214300

A_23_P24948

A_23_P250644

A_23_P312932

A_23_P32175

A_23_P334218

A_23_P3424

A_23_P350704

A_23_P352950

A_23_P354314

A_23_P356004

A_23_P37244

A_23_P383915

A_23_P38816

A_23_P411806

A_23_P414308

A_23_P416112

A_23_P419947

A_23_P518

A_23_P52410

A_23_P53467

A_23_P55149

A_23_P60166

A_23_P60271

A_23_P64404

A_23_P65918

A_23_P69573

A_23_P7005

A_23_P72961

A_23_P74115

A_23_P78835

A_23_P80974

A_23_P82839

A_23_P84118

A_23_P90032

A_23_P92225

A_23_P92467

A_23_P94795

A_23_P9603

A_23_P98910

A_23_P99292

A_24_P133488

A_24_P152345

A_24_P165423

A_24_P184799

A_24_P203056

A_24_P225616

A_24_P227121

A_24_P237804

A_24_P246926

A_24_P257759

A_24_P287941

A_24_P297182

A_24_P348203

A_24_P353794

A_24_P372189

A_24_P392109

A_24_P410797

A_24_P57631

A_24_P622186

A_24_P680947

A_24_P732106

A_24_P828949

A_24_P941912

A_32_P11764

A_32_P144018

A_32_P164215

A_32_P201329

A_32_P458096

A_32_P60065

A_32_P66364

A_32_P72341

A_32_P77989

A_32_P83049

A_32_P83256

A_32_P92783

Module #28

A_23_P120103

A_23_P121614

A_23_P127565

A_23_P129602

A_23_P151150

A_23_P154740

A_23_P15727

A_23_P160025

A_23_P163306

A_23_P170534

A_23_P202720

A_23_P203419

A_23_P205159

A_23_P212508

A_23_P253345

A_23_P253350

A_23_P300070

A_23_P302116

A_23_P309381

A_23_P332960

A_23_P348028

A_23_P35092

A_23_P354297

A_23_P355295

A_23_P36157

A_23_P364324

A_23_P369899

A_23_P373598

A_23_P374782

A_23_P387471

A_23_P411723

A_23_P41470

A_23_P417951

A_23_P51926

A_23_P5339

A_23_P55738

A_23_P62890

A_23_P70007

A_23_P70968

A_23_P75380

A_23_P83388

A_23_P88731

A_23_P99661

A_24_P190168

A_24_P193295

A_24_P263672

A_24_P281374

A_24_P299318

A_24_P320699

A_24_P325520

A_24_P335305

A_24_P353709

A_24_P360674

A_24_P406601

A_24_P411815

A_24_P42501

A_24_P47182

A_24_P788227

A_24_P932887

A_24_P93523

A_24_P935986

A_32_P101031

A_32_P120084

A_32_P170547

A_32_P207885

A_32_P29965

A_32_P32061

A_32_P34589

A_32_P74983

A_32_P89837

A_32_P98072

Module #29

A_23_P107051

A_23_P128174

A_23_P12874

A_23_P131825

A_23_P134109

A_23_P13753

A_23_P14508

A_23_P15146

A_23_P156970

A_23_P252857

A_23_P32558

A_23_P331098

A_23_P33433

A_23_P35399

A_23_P357936

A_23_P375

A_23_P40821

A_23_P432947

A_23_P43350

A_23_P50456

A_23_P66599

A_23_P73589

A_23_P79251

A_23_P93442

A_24_P118531

A_24_P238896

A_24_P323932

A_24_P451992

A_24_P64039

A_24_P88850

A_24_P941643

A_32_P186474

A_32_P226768

A_32_P31300

A_32_P62997

A_32_P84242

Module #30

A_23_P103703

A_23_P108157

A_23_P111240

A_23_P119789

A_23_P127495

A_23_P128323

A_23_P1320

A_23_P1505

A_23_P161686

A_23_P163481

A_23_P204133

A_23_P215070

A_23_P24414

A_23_P252052

A_23_P25706

A_23_P30069

A_23_P312851

A_23_P34510

A_23_P376557

A_23_P380857

A_23_P384663

A_23_P408285

A_23_P419107

A_23_P419760

A_23_P51534

A_23_P53439

A_23_P62831

A_23_P64962

A_23_P65779

A_23_P66454

A_23_P67453

A_23_P67980

A_23_P6909

A_23_P87532

A_23_P9932

A_24_P106728

A_24_P135319

A_24_P149248

A_24_P209171

A_24_P226278

A_24_P257579

A_24_P286687

A_24_P301846

A_24_P339514

A_24_P343929

A_24_P371281

A_24_P38347

A_24_P38944

A_24_P416079

A_24_P51061

A_24_P649747

A_24_P921446

A_32_P119154

A_32_P122793

A_32_P133840

A_32_P159612

A_32_P169785

A_32_P190404

A_32_P200025

A_32_P209163

A_32_P217471

A_32_P22245

A_32_P2342

A_32_P56759

A_32_P68942

Module #31

A_23_P100127

A_23_P104522

A_23_P105475

A_23_P106727

A_23_P10873

A_23_P109171

A_23_P109269

A_23_P109655

A_23_P110196

A_23_P110802

A_23_P111260

A_23_P120325

A_23_P123596

A_23_P126298

A_23_P133408

A_23_P134946

A_23_P135755

A_23_P138680

A_23_P143331

A_23_P143713

A_23_P144911

A_23_P154929

A_23_P156497

A_23_P157136

A_23_P158330

A_23_P161727

A_23_P162037

A_23_P167401

A_23_P167941

A_23_P168556

A_23_P18798

A_23_P212050

A_23_P216200

A_23_P216689

A_23_P217258

A_23_P23206

A_23_P251232

A_23_P25150

A_23_P252082

A_23_P256205

A_23_P256425

A_23_P256487

A_23_P256724

A_23_P257871

A_23_P27013

A_23_P29204

A_23_P317591

A_23_P318284

A_23_P319583

A_23_P32021

A_23_P324754

A_23_P329271

A_23_P331908

A_23_P346093

A_23_P356585

A_23_P357104

A_23_P35995

A_23_P360240

A_23_P36187

A_23_P36397

A_23_P371239

A_23_P371495

A_23_P386398

A_23_P38677

A_23_P390068

A_23_P397543

A_23_P399292

A_23_P40156

A_23_P421306

A_23_P42353

A_23_P427075

A_23_P42746

A_23_P428260

A_23_P431388

A_23_P434212

A_23_P434919

A_23_P500614

A_23_P500998

A_23_P50368

A_23_P55356

A_23_P56369

A_23_P5831

A_23_P62159

A_23_P68547

A_23_P74112

A_23_P76136

A_23_P77401

A_23_P82420

A_23_P83007

A_23_P92727

A_23_P94703

A_23_P99642

A_24_P104689

A_24_P110012

A_24_P125335

A_24_P126628

A_24_P146892

A_24_P202497

A_24_P228717

A_24_P243834

A_24_P245767

A_24_P247303

A_24_P263330

A_24_P277155

A_24_P280762

A_24_P295590

A_24_P330030

A_24_P344087

A_24_P350136

A_24_P354689

A_24_P354900

A_24_P365015

A_24_P385611

A_24_P394865

A_24_P418637

A_24_P459880

A_24_P614148

A_24_P650482

A_24_P68088

A_24_P6850

A_24_P792988

A_24_P85850

A_24_P911179

A_24_P945215

A_32_P103131

A_32_P103695

A_32_P116203

A_32_P144421

A_32_P14737

A_32_P159651

A_32_P189781

A_32_P206899

A_32_P217261

A_32_P219116

A_32_P219368

A_32_P224040

A_32_P24122

A_32_P3214

A_32_P55241

A_32_P67266

A_32_P96036

Module #32

A_23_P10025

A_23_P102950

A_23_P115064

A_23_P117602

A_23_P13364

A_23_P13425

A_23_P14072

A_23_P152406

A_23_P168211

A_23_P200203

A_23_P212500

A_23_P213832

A_23_P215875

A_23_P253434

A_23_P27315

A_23_P31873

A_23_P325690

A_23_P37399

A_23_P393034

A_23_P41674

A_23_P418006

A_23_P58642

A_23_P60599

A_23_P60990

A_23_P62021

A_23_P62115

A_24_P133475

A_24_P15621

A_24_P254437

A_24_P317762

A_24_P365365

A_24_P382319

A_24_P64442

A_24_P87036

A_24_P876864

A_32_P10327

A_32_P110156

A_32_P175539

A_32_P37721

Module #33

A_23_P109452

A_23_P110957

A_23_P143047

A_23_P165608

A_23_P203957

A_23_P21560

A_23_P256190

A_23_P257516

A_23_P301051

A_23_P332399

A_23_P358597

A_23_P37068

A_23_P373992

A_23_P381992

A_23_P411335

A_23_P416894

A_23_P49136

A_23_P51690

A_23_P83328

A_23_P86623

A_24_P112160

A_24_P179400

A_24_P235870

A_24_P249253

A_24_P276888

A_24_P27977

A_24_P330684

A_24_P334130

A_24_P345679

A_24_P363583

A_24_P382187

A_24_P39508

A_24_P406693

A_24_P41794

A_24_P76521

A_24_P8454

A_24_P924721

A_24_P933418

A_32_P192823

A_32_P219963

A_32_P7516

A_32_P80741

A_32_P92415

A_32_P93144

Module #34

A_23_P108280

A_23_P132560

A_23_P137689

A_23_P145846

A_23_P157600

A_23_P2233

A_23_P258887

A_23_P259207

A_23_P28334

A_23_P384551

A_23_P386478

A_23_P409168

A_23_P423074

A_23_P4679

A_23_P47735

A_23_P66635

A_23_P72059

A_23_P73220

A_23_P89981

A_23_P93360

A_23_P95634

A_24_P122732

A_24_P343621

A_24_P39195

A_24_P392505

A_24_P532589

A_24_P565110

A_24_P58597

A_24_P589001

A_24_P89987

A_24_P935252

A_32_P175557

A_32_P184916

A_32_P196263

A_32_P27271

A_32_P36694

A_32_P83915

A_32_P99171

Module #35

A_23_P101093

A_23_P101351

A_23_P102071

A_23_P1029

A_23_P103561

A_23_P104199

A_23_P106362

A_23_P112241

A_23_P116942

A_23_P11862

A_23_P122216

A_23_P123276

A_23_P124742

A_23_P126825

A_23_P129246

A_23_P129925

A_23_P131866

A_23_P132159

A_23_P132718

A_23_P133694

A_23_P135977

A_23_P138194

A_23_P139600

A_23_P14083

A_23_P143016

A_23_P145096

A_23_P145408

A_23_P151405

A_23_P153050

A_23_P153197

A_23_P153958

A_23_P158829

A_23_P159907

A_23_P160934

A_23_P161624

A_23_P164047

A_23_P167040

A_23_P167389

A_23_P168828

A_23_P169039

A_23_P169189

A_23_P18966

A_23_P19134

A_23_P19852

A_23_P200138

A_23_P200310

A_23_P201628

A_23_P202881

A_23_P203351

A_23_P206598

A_23_P208493

A_23_P209183

A_23_P209954

A_23_P211007

A_23_P212552

A_23_P21324

A_23_P213944

A_23_P214176

A_23_P215341

A_23_P216836

A_23_P217015

A_23_P217049

A_23_P217379

A_23_P22422

A_23_P25097

A_23_P252106

A_23_P25293

A_23_P254626

A_23_P254888

A_23_P255104

A_23_P255263

A_23_P255672

A_23_P27515

A_23_P27947

A_23_P28105

A_23_P28169

A_23_P315815

A_23_P316741

A_23_P318115

A_23_P319859

A_23_P33256

A_23_P335428

A_23_P338919

A_23_P339818

A_23_P340909

A_23_P342067

A_23_P344451

A_23_P349676

A_23_P356554

A_23_P370682

A_23_P374862

A_23_P376449

A_23_P388146

A_23_P3963

A_23_P40049

A_23_P40240

A_23_P403335

A_23_P408455

A_23_P415443

A_23_P41765

A_23_P41917

A_23_P420196

A_23_P42575

A_23_P426021

A_23_P43810

A_23_P48637

A_23_P51085

A_23_P54626

A_23_P55091

A_23_P5601

A_23_P56630

A_23_P57306

A_23_P61447

A_23_P63038

A_23_P63390

A_23_P65481

A_23_P65963

A_23_P67725

A_23_P70785

A_23_P70915

A_23_P71904

A_23_P73097

A_23_P76015

A_23_P76914

A_23_P80382

A_23_P81805

A_23_P85716

A_23_P88411

A_23_P92441

A_23_P94422

A_23_P98159

A_23_P9823

A_24_P123155

A_24_P14595

A_24_P175435

A_24_P17870

A_24_P179504

A_24_P183150

A_24_P206305

A_24_P211565

A_24_P213325

A_24_P218979

A_24_P220454

A_24_P234196

A_24_P234871

A_24_P24819

A_24_P258073

A_24_P264293

A_24_P264644

A_24_P274795

A_24_P280873

A_24_P283189

A_24_P291814

A_24_P314159

A_24_P322474

A_24_P32252

A_24_P330234

A_24_P334361

A_24_P354715

A_24_P365975

A_24_P370670

A_24_P376391

A_24_P378987

A_24_P403244

A_24_P46417

A_24_P46689

A_24_P557479

A_24_P561165

A_24_P584992

A_24_P58894

A_24_P655888

A_24_P69439

A_24_P755069

A_24_P84419

A_24_P923612

A_24_P937405

A_24_P945113

A_24_P97703

A_32_P108254

A_32_P117185

A_32_P131367

A_32_P174978

A_32_P185229

A_32_P196047

A_32_P210642

A_32_P231302

A_32_P2452

A_32_P26443

A_32_P313405

A_32_P37974

A_32_P52076

A_32_P64570

A_32_P86318

A_32_P877

A_32_P9753

Module #36

A_23_P10559

A_23_P109072

A_23_P113553

A_23_P120125

A_23_P121374

A_23_P123022

A_23_P123943

A_23_P130158

A_23_P139919

A_23_P145175

A_23_P148984

A_23_P149892

A_23_P151133

A_23_P152791

A_23_P153562

A_23_P154217

A_23_P156198

A_23_P158318

A_23_P159255

A_23_P159986

A_23_P161297

A_23_P16409

A_23_P200843

A_23_P201837

A_23_P202138

A_23_P20427

A_23_P204879

A_23_P207125

A_23_P210811

A_23_P215265

A_23_P215479

A_23_P250516

A_23_P255345

A_23_P258234

A_23_P27256

A_23_P28664

A_23_P301521

A_23_P30870

A_23_P310421

A_23_P312132

A_23_P324340

A_23_P330561

A_23_P33277

A_23_P349147

A_23_P350574

A_23_P354694

A_23_P354805

A_23_P358628

A_23_P360964

A_23_P371966

A_23_P389250

A_23_P392575

A_23_P39665

A_23_P40295

A_23_P407840

A_23_P408376

A_23_P409462

A_23_P41888

A_23_P42282

A_23_P425332

A_23_P430658

A_23_P43276

A_23_P45970

A_23_P47247

A_23_P55649

A_23_P6099

A_23_P62764

A_23_P63209

A_23_P63541

A_23_P7361

A_23_P76774

A_23_P78405

A_23_P79108

A_23_P86171

A_23_P92370

A_23_P97157

A_23_P9883

A_24_P11384

A_24_P116587

A_24_P118196

A_24_P135461

A_24_P145134

A_24_P148261

A_24_P170717

A_24_P179569

A_24_P194881

A_24_P195454

A_24_P196851

A_24_P20120

A_24_P203418

A_24_P205994

A_24_P270279

A_24_P272313

A_24_P316430

A_24_P342178

A_24_P388860

A_24_P406006

A_24_P4373

A_24_P48177

A_24_P59607

A_24_P602871

A_24_P64241

A_24_P725630

A_24_P887615

A_24_P913056

A_24_P921264

A_24_P933319

A_32_P135890

A_32_P164917

A_32_P170206

A_32_P171143

A_32_P183022

A_32_P212058

A_32_P225328

A_32_P230547

A_32_P3783

A_32_P4199

A_32_P66881

A_32_P70273

A_32_P760762

A_32_P83776

A_32_P87531

Module #37

A_23_P115316

A_23_P118834

A_23_P119015

A_23_P12082

A_23_P124024

A_23_P128319

A_23_P129476

A_23_P13222

A_23_P132809

A_23_P134085

A_23_P136721

A_23_P137935

A_23_P152909

A_23_P162547

A_23_P168531

A_23_P208310

A_23_P213298

A_23_P216556

A_23_P318300

A_23_P33511

A_23_P344531

A_23_P369328

A_23_P377434

A_23_P424

A_23_P44195

A_23_P49192

A_23_P50815

A_23_P51951

A_23_P54622

A_23_P62857

A_23_P64837

A_23_P65442

A_23_P67529

A_23_P70938

A_23_P73540

A_23_P73982

A_23_P74349

A_23_P86470

A_24_P12401

A_24_P157424

A_24_P174793

A_24_P187174

A_24_P193648

A_24_P2463

A_24_P246573

A_24_P255218

A_24_P300394

A_24_P336417

A_24_P380734

A_24_P383609

A_24_P396375

A_24_P666340

A_24_P71904

A_24_P766716

A_24_P82106

A_24_P84130

A_24_P96403

A_32_P116058

A_32_P122492

A_32_P122754

A_32_P130536

A_32_P152986

A_32_P210202

A_32_P219660

A_32_P224727

A_32_P32739

A_32_P74942

A_32_P83845

A_32_P97169

Module #38

A_23_P101505

A_23_P106145

A_23_P133691

A_23_P154507

A_23_P257993

A_23_P26024

A_23_P310

A_23_P366394

A_23_P366812

A_23_P373119

A_23_P401580

A_23_P46315

A_23_P47682

A_23_P500010

A_23_P50897

A_23_P65618

A_23_P88691

A_23_P92222

A_23_P93349

A_23_P96568

A_23_P98580

A_24_P115199

A_24_P130962

A_24_P171075

A_24_P191047

A_24_P210829

A_24_P312058

A_24_P379104

A_24_P472055

A_24_P53519

A_24_P568645

A_24_P913146

A_24_P93321

A_32_P201521

A_32_P208991

A_32_P98227

Module #39

A_23_P106675

A_23_P109026

A_23_P127824

A_23_P129188

A_23_P135385

A_23_P144778

A_23_P157117

A_23_P20480

A_23_P209778

A_23_P330611

A_23_P363769

A_23_P422268

A_23_P48198

A_23_P8253

A_24_P135344

A_24_P160466

A_24_P211064

A_24_P309645

A_24_P316965

A_24_P331830

A_24_P415012

A_24_P625898

A_24_P78531

A_24_P911973

A_24_P928052

A_32_P104478

A_32_P141682

A_32_P227110

A_32_P515920

A_32_P56713

Module #40

A_23_P103486

A_23_P111804

A_23_P118038

A_23_P12199

A_23_P12746

A_23_P140029

A_23_P155009

A_23_P162579

A_23_P163390

A_23_P203882

A_23_P206945

A_23_P208880

A_23_P214969

A_23_P252681

A_23_P253145

A_23_P25964

A_23_P306964

A_23_P311087

A_23_P315571

A_23_P350005

A_23_P369966

A_23_P37088

A_23_P374149

A_23_P379614

A_23_P381368

A_23_P384355

A_23_P388812

A_23_P3956

A_23_P400794

A_23_P404259

A_23_P406385

A_23_P49972

A_23_P500381

A_23_P51986

A_23_P52198

A_23_P58117

A_23_P61487

A_23_P62387

A_23_P7679

A_23_P81392

A_23_P98002

A_23_P99802

A_24_P137376

A_24_P25040

A_24_P304071

A_24_P304439

A_24_P339126

A_24_P357169

A_24_P397515

A_24_P46130

A_24_P707530

A_24_P711050

A_24_P712350

A_24_P816844

A_24_P913431

A_24_P914479

A_24_P926115

A_32_P110505

A_32_P140049

A_32_P178945

A_32_P181107

A_32_P210168

A_32_P224522

A_32_P5205

A_32_P70818

**Supplementary figures**


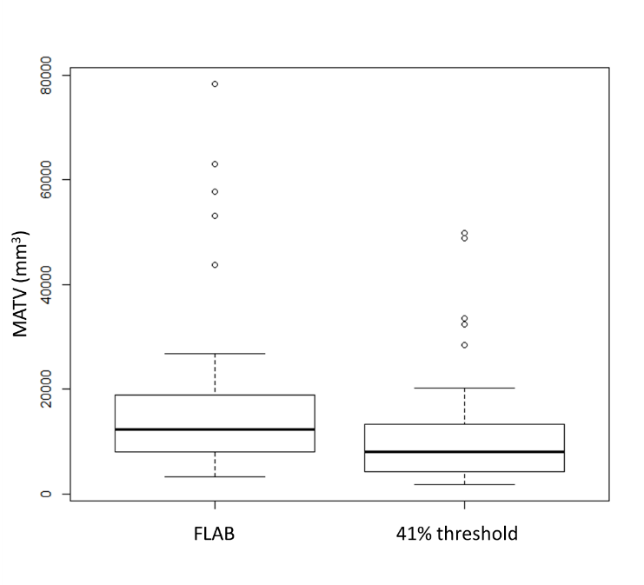


**Supplemental figure A:** Boxplots showing the MATVs obtain using FLAB and a threshold at 41% of the SUV_max_.


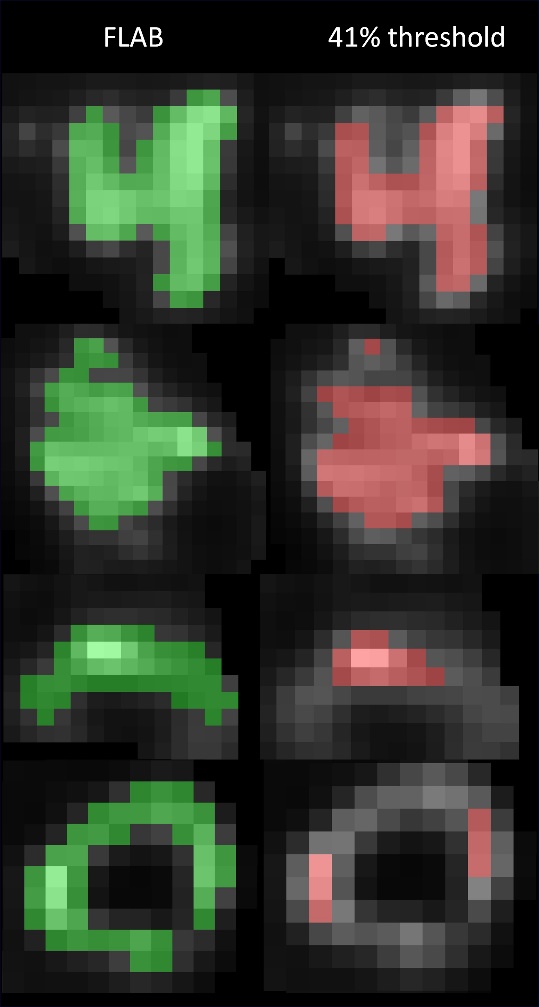


**Supplemental figure B:** 4 examples of MATVs obtain with FLAB in green (on the left) and with a threshold at 41% of the SUV_max_ (on the right).

**
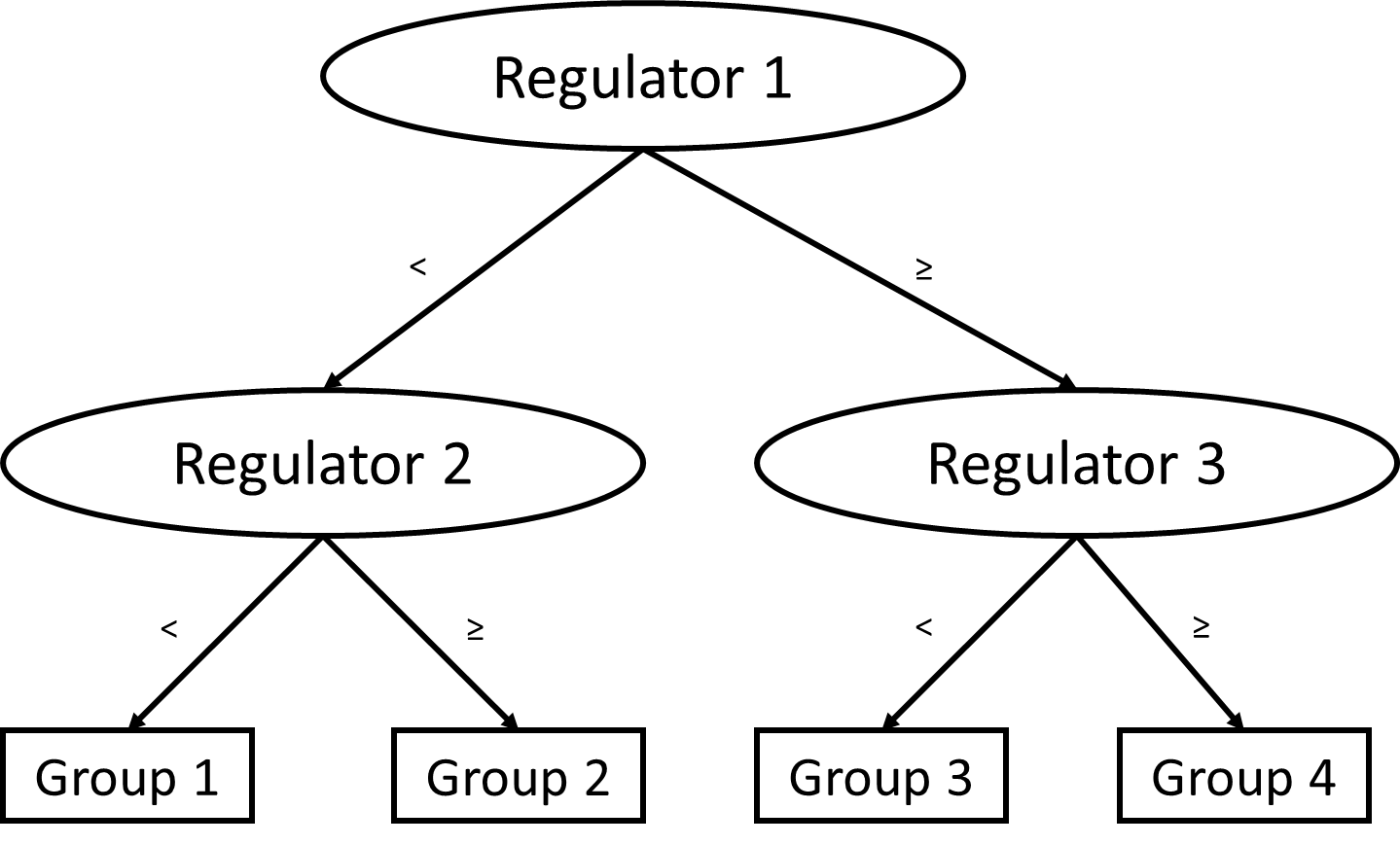
**

**Supplemental figure C:** Graphical view of the way how PET regulators were combined to create subgroups of patients.

**Supplemental figure D:** Percentage of probes corresponding to genes described in the Reactome Pathway Database (in green), gene with unknown function (in red) and probes relatives to ncRNA (in purple) for the 40 modules.
